# Thermodynamic Origin‐Based In Situ Electrochemical Construction of Reversible p‐n Heterojunctions for Optimal Stability in Potassium Ion Storage

**DOI:** 10.1002/advs.202308582

**Published:** 2024-03-13

**Authors:** Wei‐Wen Shen, Yi‐Yen Hsieh, Yi‐Chun Yang, Kai‐Yuan Hsiao, Ming‐Yen Lu, Chi Wei Chou, Hsing‐Yu Tuan

**Affiliations:** ^1^ Department of Chemical Engineering National Tsing Hua University Hsinchu 30013 Taiwan; ^2^ Department of Materials Science and Engineering National Tsing Hua University Hsinchu 30013 Taiwan

**Keywords:** heterojunction, in situ construction, potassium‐ion batteries, reversible p‐n junction

## Abstract

Heterojunctions in electrode materials offer diverse improvements during the cycling process of energy storage devices, such as volume change buffering, accelerated ion/electron transfer, and better electrode structure integrity, however, obtaining optimal heterostructures with nanoscale domains remains challenging within constrained materials. A novel in situ electrochemical method is introduced to develop a reversible CuSe/PSe p‐n heterojunction (CPS‐h) from Cu_3_PSe_4_ as starting material, targeting maximum stability in potassium ion storage. The CPS‐h formation is thermodynamically favorable, characterized by its superior reversibility, minimized diffusion barriers, and enhanced conversion post K^+^ interaction. Within CPS‐h, the synergy of the intrinsic electric field and P‐Se bonds enhance electrode stability, effectively countering the Se shuttling phenomenon. The specific orientation between CuSe and PSe leads to a 35° lattice mismatch generates large space at the interface, promoting efficient K ion migration. The Mott‐Schottky analysis validates the consistent reversibility of CPS‐h, underlining its electrochemical reliability. Notably, CPS‐h demonstrates a negligible 0.005% capacity reduction over 10,000 half‐cell cycles and remains stable through 2,000 and 4,000 cycles in full cells and hybrid capacitors, respectively. This study emphasizes the pivotal role of electrochemical dynamics in formulating highly stable p‐n heterojunctions, representing a significant advancement in potassium‐ion battery (PIB) electrode engineering.

## Graphical abstract

An electrochemical method is presented for creating a stable, reversible CuSe/PSe (CPS‐h) p‐n heterojunction from Cu_3_PSe_4_. This approach ensures thermodynamic favorability of CPS‐h, leading to enhanced potassium‐ion storage stability, reduced diffusion barriers, and improved conversion efficiency. Emphasizing the importance of electrochemical processes, this advancement represents a significant step forward in the development of potassium‐ion battery electrodes.

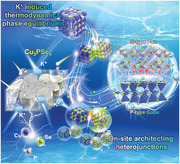

## 1 Introduction

Potassium‐ion batteries (PIBs) are gaining attention for their chemical and economic advantages. Chemically, they have a low K^+^/K potential of −2.88 V in carbonate electrolytes, indicating high energy density and minimal risk of K plating. These batteries also benefit from the fast diffusion rate of K‐ions in carbonate electrolytes.^[^ ^1^, ^2^ ^]^ Economically, potassium is abundant and globally available.^[^ ^3^, ^4^ ^]^ Conversion‐type materials are widely researched as PIB anodes because of their potential for higher specific capacity through multi‐electron conversion reactions. However, they face challenges like significant volume changes during charging and discharging due to large‐sized K^+^ reactions,^[^ ^5^ ^]^ leading to structural issues like aggregation and pulverization,^[^ ^6^, ^7^ ^]^ and resulting in poor Coulombic efficiency and quick capacity loss.^[^ ^8^, ^9^, ^10^ ^]^ Optimizing these anodes can be achieved through methods such as site doping/modification,^[^ ^11^ ^]^ phase modulation, growth morphology control, and heterostructure construction.^[^ ^12^, ^13^, ^14^ ^]^ Recent advancements in energy storage have highlighted the significance of functionally oriented heterostructures.^[^ ^15^, ^16^, ^17^, ^18^, ^19^, ^20^, ^21^, ^22^ ^]^ When two semiconductors with distinct energy bands come into contact, thermodynamic equilibrium is achieved, leading to opposing space charge regions and intrinsic electric fields. This phenomenon offers multiple advantages: 1) The inherent electric field and concentration gradient enhance electrochemical reaction kinetics, accelerating ion migration.^[^ ^21^ ^]^ 2) Quantum tunneling permits certain electrons, even those below energy barriers, to access the interface state. 3) During charge and discharge, various electrochemically active reaction intermediates form. A lower potential intermediate can counteract the volume change of a higher one, thus increasing cycle stability.^[^ ^23^ ^]^ The p‐n junction is a common heterojunction type. Given the excess holes in the p‐type and the electrons in the n‐type, charges move at the interface until equilibrium, leading to an internal electric field that boosts ion migration.^[^ ^24^ ^]^ An illustration of this is Hsieh et al.’s work on mixed‐dimensional Bi_2_S_3_/Bi_2_Se_3_ vdWH topological p‐n heterostructures, which improve K^+^ diffusion, electronic transfer, and electrolyte penetration.^[^ ^25^ ^]^ Besides the conventional p‐n junctions, energy storage also sees the use of Schottky heterojunctions, formed when metals and semiconductors interact. Typically, n‐type semiconductors have a Fermi level surpassing that of metals. This difference causes electrons to migrate, establishing an electric field and a depletion region at the junction. A notable application is Sun et al.’s utilization of Schottky junctions to activate silicon carbide for high‐performance lithium‐ion battery anodes.^[^ ^26^ ^]^ Lastly, the interfacially coupled structure creates a Schottky junction enhanced by Si‐C interactions. While depletion regions can form in homogeneous heterojunctions like n‐n or p‐p, their electron‐hole recombination rate is lower than that of the p‐n type, making them rarer in battery technology.

Optimal heterojunctions must be nanoscale, facilitating numerous heterostructures within constrained materials for enhanced cycling. For uniform ion/electron transport, a steady electric field and unique inorganic interfaces are required. The growth mode during heterogeneous deposition is guided by the thermodynamic equation Δ *G_S_* = γ_1_  − γ_2_ + γ_1,2_. In this, γ_1_ and γ_2_ represent the surface energies of the materials, and γ_1,2_ denotes the interfacial energy between them.^[^ ^27^ ^]^ Chemical vapor deposition is favored in semiconductor production for its uniform growth.^[^ ^28^, ^29^, ^30^ ^]^ However, its use in alkali metal ion energy storage limits performance due to its one dimensional (1D) size control, unsuitable for three dimensional (3D) nanocrystals. Thus, alternative methods are needed for heterojunction construction in energy storage. Using templates, precursors were reacted by introducing a third element, like sulfur^[^ ^31^ ^]^ or selenium,^[^ ^32^ ^]^ and phosphorous^[^ ^33^ ^]^ through gas or liquid phase methods. Heterojunctions were obtained by combining various dimensions (e.g., zero dimensional (0D)− two dimensional (2D),^[^ ^34^ ^]^ 2D−2D,^[^ ^35^ ^]^ 0D−3D,^[^ ^36^ ^]^ and 2D−3D).^[^ ^37^ ^]^ These multi‐step procedures result in resource waste and reduced productivity. On the other hand, an uncomplicated has been proposed to construct heterojunction. Susaria et al. converted a quaternary alloy into a 2D binary alloy heterojunction using thermal annealing,^[^ ^38^ ^]^ indicating that multivariate compounds can form in situ heterostructures through certain methods.

**Scheme 1:**
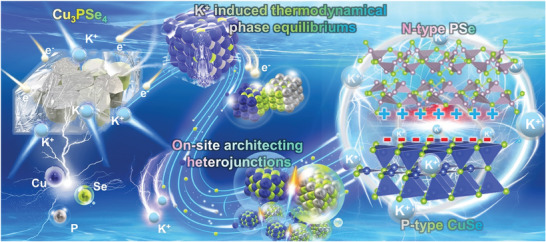
Schematic illustration of in situ constructing CuSe/PSe p‐n heterojunction from Cu_3_PSe_4_ after electrochemical reaction with K ions.

In this study, we show that while the insertion/extraction of potassium ions for ternary Cu_3_PSe_4_, CuSe and PSe co‐existed in the system in a thermodynamically favorable form, and in situ constructed CuSe/PSe p‐n heterojunction (denoted as CPS‐h) plane in a molecularly engineered form (**Scheme** **1**). The CPS‐h reveals superior kinetics and electrochemical performance as a potassium ion anode material. Experiment and density functional theory (DFT) calculations show that CPS‐h exhibit the following superior properties and performances: 1) long‐term reversible p‐n heterojunction formation, causing direct migration of electrons and K ions between CuSe and PSe, and achieving spatially optimized distribution of charged species; 2) alloyed P−Se produces strong chemical bonds, which can anchor Se during cycling and suppress the shuttling effect of chalcogenides; 3) large‐scale construction of heterostructures with the lowest potassium on the interface The ion diffusion resistance and the most excellent potassium ion adsorption capacity accelerate the ion/electron transfer kinetics; 4) the half‐cell cycle of CPS‐h/G reaches an ultra‐stable 10000 times, and the continuous operation time exceeds 6974 h, and successfully reached 2000 cycles in the full battery system.

## 2 Results and Discussion

### 2.1 Material Characterization of Cu_3_PSe_4_

**Figure 1:**
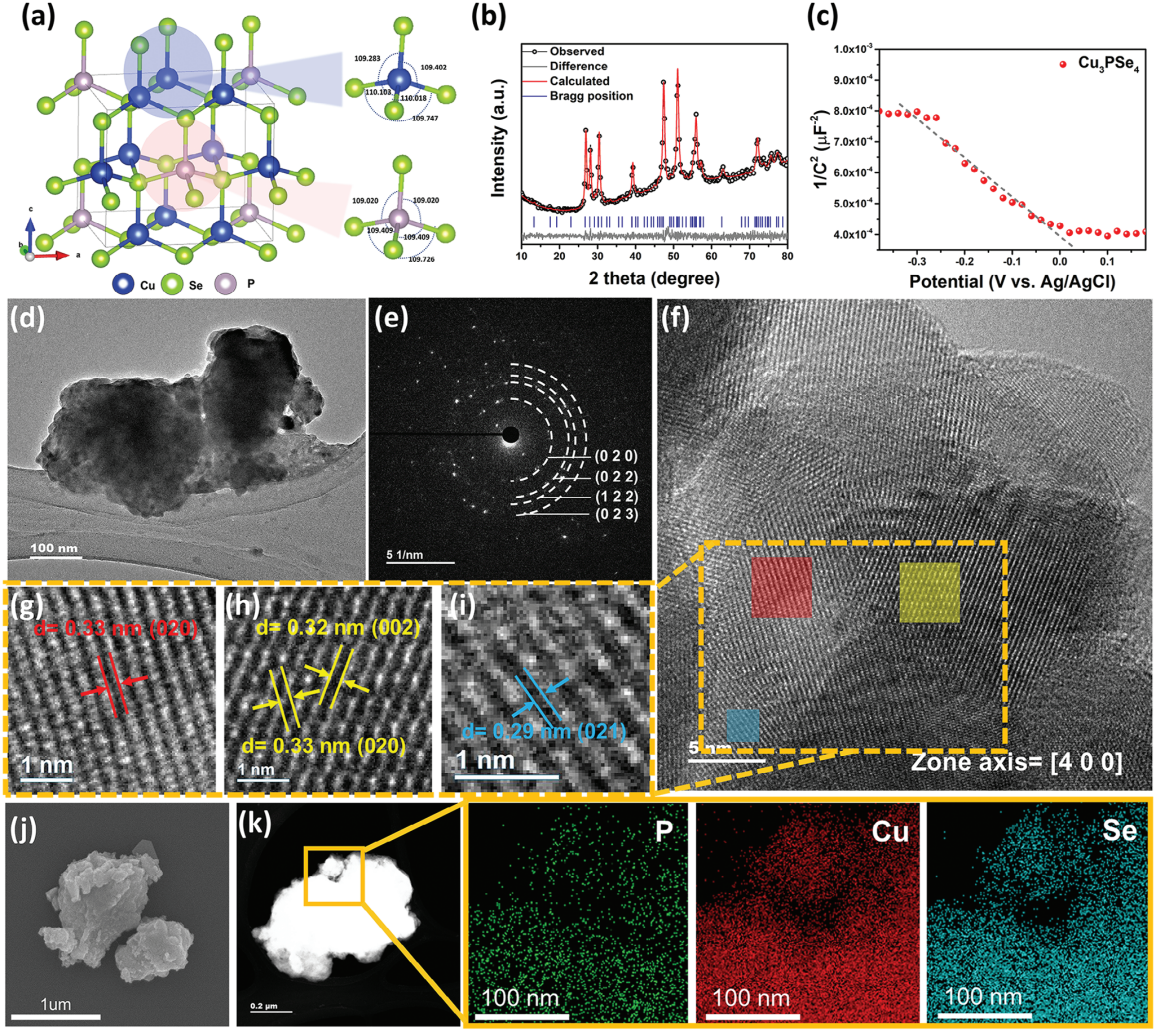
Material characterization of Cu_3_PSe_4_. a) The crystal structure of a unit cell. b) Powder XRD patterns with Rietveld refinement. c) Mott‐Schottky plot. d) TEM images. e) SAED ring pattern. f–i) HRTEM images. j) SEM image. k) EDS element mapping images.

In the Cu_3_−V−VI_4_ chalcogenides (V = P, As, Sb, Bi, VI = S, Se, Te), each anion must satisfy the octet rule, maintaining eight valence electrons by bonding with three Cu atoms and one group V atom. This leads to four possible crystal structures for Cu_3_−V−VI_4_: enargite (space group Pmn2_1_), wurtzite‐PMCA (space group P6_3_mc), famatinite (space group I $\bar{4}$ 2m), and zinc blend‐PMCA (space group P $\bar{4}$ 3m).^[^ ^39^ ^]^ We have synthesized Cu_3_PSe_4_ with an enargite crystal structure for the first time via high‐energy ball milling. As shown in **Figure** **1**a, Cu_3_PSe_4_ exhibits a tetrahedral structure where each atom connects to four nearest neighbors. Notably, each monovalent Cu^+^ and pentavalent P^5+^ cation is coordinated with four Se^2−^ anions, forming [CuSe_4_] and [PSe_4_] tetrahedra.^[^ ^40^ ^]^ We synthesized Cu_3_PSe_4_ powder through a process where Cu_3_P (Figure S1, Supporting Information) and Se powder were combined in a 1:4 molar ratio and ball milled under an argon atmosphere. The resulting compound crystal structure was confirmed by powder X‐ray diffraction (XRD) analysis. As shown in Figure 1b, all diffraction peaks matched the orthorhombic structure of Pmn2_1_ (PDF 04‐006‐8347), with lattice parameters of a = 7.697 Å, b = 6.661 Å, and c = 6.381 Å. The Rietveld refinement curve showed no extraneous peaks. The five strongest diffraction peaks at 26.73°, 27.94°, 30.23°, 47.22°, and 50.89° were attributed to (020), (002), (021), (230), and (023) crystal planes, respectively.

We conducted Mott‐Schottky measurements using a three‐electrode electrochemical system, shown in Figure 1c. The negative slope of the Mott‐Schottky plot signifies Cu_3_PSe_4_ p‐type semiconductor characteristic,^[^ ^41^ ^]^ aligning with earlier reports.^[^ ^42^, ^43^, ^44^ ^]^ To improve electrochemical performance stability, we applied a graphite coating to Cu_3_PSe_4_, forming a Cu_3_PSe_4_/G composite (Figure S2, Supporting Information). The Raman spectrum (Figure S3a, Supporting Information) reveals a relative intensity ratio of 1.22 for the D band (1350 cm^−1^) to the G band (1580 cm^−1^), verifying the formation of the composite. The D band, attributed to the breathing mode of sp^2^ hybridized carbon, becomes active due to disorder and edges in polycrystalline graphite, while it's inactive in symmetric sites in well‐crystallized graphite. The G band, attributed to the stretching mode of sp^2^ hybridized carbon, is ≈1580 cm^−1^.^[^ ^45^ ^]^ Thermogravimetric analysis (Figure S3b, Supporting Information) shows the Cu_3_PSe_4_/G composite possesses high thermal stability up to 340 °C. X‐ray photoelectron spectroscopy (XPS) was used to confirm Cu_3_PSe_4_ composition and chemical state. The Cu 2p high‐resolution spectrum divides into two peaks: prominent peaks at 932.6 and 952.5 eV correspond to Cu 2p_3/2_ and Co 2p_1/2_ of Cu^+^. Unlike divalent copper (Cu^2+^) that presents satellite peak signals due to multiple splits in a partially filled d‐shell, the monovalent copper (Cu^+^) with its fully filled d‐shell, doesn't cause multiple splitting in the Cu 2p spectrum (Figure S4a, Supporting Information).^[^ ^46^ ^]^ In the P 2p spectrum (Figure S4b, Supporting Information), peaks at 131.4 and 133.5 eV represent P 2p_3/2_ and P 2p_1/2_, and peaks at 135.7 eV indicate surface partially oxidized P−O.^[^ ^47^ ^]^ The broad peak at 138.5 eV corresponds to the Se LMM_2_ signal in Cu_3_PSe_4_. In the Se 3d spectrum (Figure S4c, Supporting Information), Se 3d_5/2_ and Se 3d_3/2_ are at 54.7 and 55.5 eV. Due to the high‐energy ball milling method, we can mass produce Cu_3_PSe_4_ but with irregular morphology and size variation (Figure 1d,j). Selected‐area electron diffraction (SAED) (Figure 1e) uncovers four rings corresponding to (020), (022), (122), and (023) planes of Cu_3_PSe_4_, agreeing with XRD interlayer spacings. The high‐resolution transmission electron microscopy (HRTEM) image (zone axis‐ [400]) reveals lattice fringes of 0.33 nm (Figure 1g), 0.32 nm (Figure 1h), and 0.29 nm (Figure 1i), corresponding to the (020), (002), and (021) crystal planes of Cu_3_PSe_4_. Energy dispersive spectroscopy (EDS) analysis (Figure 1k) shows that Cu, P, and Se are present and uniformly distributed in Cu_3_PSe_4_. Particularly, Cu and Se signals exceed that of P, consistent with the molar ratio in Cu_3_PSe_4_.

## 3 The Rationality of In Situ Construction of CuSe/PSe Co‐Presence Phase

**Figure 2:**
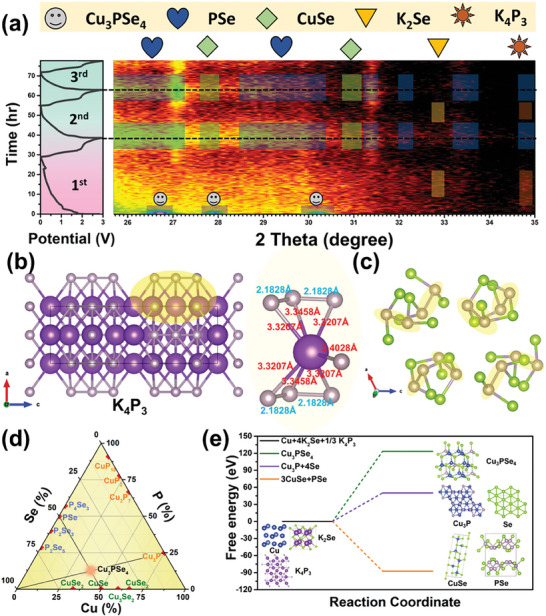
Mechanism derivation. a) In situ XRD patterns of Cu_3_PSe_4_ half‐cell for first three cycles. b) The crystal structure of K_4_P_3_ and its segment of the (100) plane that made up of recurring K−P (3.3207 and 3.4028 Å) and P−P (2.1828 Å) bonds. c) The crystal structure of PSe with lots of P−P bonds existing. d) Cu−P−Se phase diagram according to different molar ratio. (Cu_3_P−4Se, 3CuSe−PSe, and the intercept is Cu_3_PSe_4_) e) The calculation of reaction free energy change based on the species at fully discharged state (Cu, K_4_P_3_, and K_2_Se), the predicted reaction products are Cu_3_PSe_4_, Cu_3_P−4Se, and 3CuSe−PSe, respectively.

In electrochemistry, the occurrence of an electrochemical reaction is governed by thermodynamics as the following equation: Δ*G*  =  Δ*G* ^0^ + *RT* ln*K*, where K relates to the balance of reactants and products. As reactions proceed, Gibbs free energy decreases due to the higher reactant concentration. In alkali ion batteries, conversion‐type materials (MXs, X = P, S, Se, Te) undergo intricate multi‐electron transfer reactions.^[^ ^48^ ^]^ The conversion reaction begins with alkali ion intercalation, transitioning through bond alterations to produce K_x_X and M^0^.^[^ ^49^ ^]^ Each step follows a path of minimal energy change.^[^ ^50^ ^]^ Advanced techniques like in situ XRD,^[^ ^51^ ^]^ TEM,^[^ ^52^ ^]^ and Raman^[^ ^53^ ^]^ are essential to decode this complex mechanism. These methods allow to detect reaction mechanisms through changes in peak positions or lattice structures. Notably, early in situ XRD patterns show that Cu_3_PSe_4_ doesn't revert to its original phase after a 3 V discharge/charge cycle (**Figure** **2**a). Upon first discharge to 0.01 V, a weak signal at 32.9° was detected, corresponding to a low crystallinity K_2_Se diffraction peak, and a K_4_P_3_ diffraction signal at 34.6°. This indicates that Cu_3_PSe_4_ mainly produces K_2_Se and K_4_P_3_ via conversion reactions during discharge, consistent with prior findings on transition metal phosphorus chalcogenides. Notably, during the initial charge to 3 V, diffraction signals at 27.1° and 31.4° differ significantly from the open circuit voltage (OCV), followed by a series of signals indicating reversible phases in the next two cycles. Figure 2b represents the unit cell of K_4_P_3_. A segment of the (100) plane was analyzed, primarily made up of recurring K−P (3.3207 and 3.4028 Å) and P−P (2.1828 Å) bonds. Given that the K−P bond is longer than the P−P bond, it is inferred that depotassiation commences with the cleavage of potassium ions from this location. As per the unit cell of the PSe structure in Figure 2c, numerous P−P and P−Se bonds exist. During K_2_Se depotassiation, abundant Se resources are released and tend to form PSe by combining with remaining P−P bonds in K_4_P_3_. Given the fourfold content of Se to P in this system, excess Se tends to form copper selenide with Cu. Notably, current Cu and Se quantities match, suggesting a 1:1 molar ratio in the resulting copper selenide, CuSe. The comparison of PSe crystal structure (PDF 04‐007‐1805) and the in situ XRD pattern at 3 V charging reveals diffraction signals at 26.72°−27.15°, 28.66°−30.82°, 31.4°, 32°, 33.3°−33.7°, and 34.86° originate from PSe diffraction peaks. CuSe signals were detected at 27.85° and 31.07° (PDF 01‐086‐1240). The results of ex situ XRD are similar to those of in situ XRD (Figure S5, Supporting Information). Redox reactions typically involve molecular splitting into multiple products or internal atomic recombination. These reactions predominantly entail the formation and disruption of chemical bonds to bring about change. Importantly, these processes don't alter the nucleus but concern only the outer electron cloud of atoms. As per the law of conservation of mass, the atom types remain consistent before and after the reaction. We utilize Figure 2d to illustrate how a ternary material, synthesized from two binary materials, in situ regenerates into two other binary materials. We prepare Cu_3_PSe_4_ powder with a Cu_3_P/Se ratio of 1/4. The coexistence of CuSe/PSe in the in situ XRD pattern charged to 3 V suggests that the intersection of two equilibrium lines is the ternary material, Cu_3_PSe_4_. Combining possible compound compositions with theoretical calculations of free energy changes of potential products after full depotassiation allows us to infer possible products post‐depotassiation. Given that the ternary material first intercalates potassium to produce K_2_Se, K_4_P_3_, and Cu, we consider the dominant reactants to be the products after potassiation. We regard the potential depotassiation products as 1) Cu_3_PSe_4_, 2) Cu_3_P−4Se, and 3) 3CuSe−PSe.

(1) $$4K_{2}Se+\frac{1}{3}K_{4}P_{3}+3Cu\to Cu_{3}PSe_{4}$$

(2) $$4K_{2}Se+\frac{1}{3}K_{4}P_{3}+3Cu\to Cu_{3}P+4Se$$

(3) $$4K_{2}Se+\frac{1}{3}K_{4}P_{3}+3Cu\to 3CuSe+PSe$$

Due to the dipole moments, the K−P bond cleaves first in oxidation. Forming PSe avoids the higher energy P−P bond breakage. Typically, 3CuSe−PSe has the lowest energy, while Cu_3_P−4Se sits at intermediate energy. Since the Cu_3_PSe_4_ unit cell only contains [CuSe_4_] and [PSe_4_] tetrahedrons, forming Cu_3_PSe_4_ from K_4_P_3_ requires more energy to cleave the P−P bond, thus rendering the reaction less likely. According to Hess's law, if the delta H of a product is less than that of a reactant, the enthalpy change of the reaction will be negative.^[^ ^54^ ^]^ According to the second law of thermodynamics, the reaction will proceed spontaneously without the need for additional energy input like heating or potential.^[^ ^55^ ^]^ This also confirms that Cu_3_PSe_4_ can spontaneously undergo a synergetic chemical reaction. In the redox process, the cleavage and formation of chemical bonds happen simultaneously. As the K−P bond cleaves, it bonds immediately with Se to form PSe, generating a stable PSe phase. Simultaneously, Cu combines with other Se to form three moles of CuSe. This explains why, after complete depotassiation, PSe and CuSe with the lowest free energy form, rather than ternary compounds or other combinations. Given the criterion of minimal free energy change, CuSe and PSe are the most preferred products when charging back to 3 V (Figure 2e). For the calculation of reaction free energy change is by the following definition:^[^ ^56^ ^]^

(4) $$E_{r1}=\;\pm \left[\left(E_{K_{2}Se}+E_{K_{4}P_{3}}+\mu_{Cu}\right)-\left(E_{Cu_{3}PSe_{4}}\right)\right]$$

(5) $$E_{r2}=\;\pm \left[\left(E_{K_{2}Se}+E_{K_{4}P_{3}}+\mu_{Cu}\right)-\left(E_{Cu_{3}P}+\mu_{Se}\right)\right]$$

(6) $$E_{r3}=\;\pm \left[\left(E_{K_{2}Se}+E_{K_{4}P_{3}}+\mu_{Cu}\right)-\left(E_{CuSe}+E_{PSe}\right)\right]$$

where E_r_ is the reaction free energy change, E_i_ and µ_i_ are the total energy and the chemical potential of species i, respectively.

### 3.1 The Ex Situ Characterizations of CPS‐h

**Figure 3:**
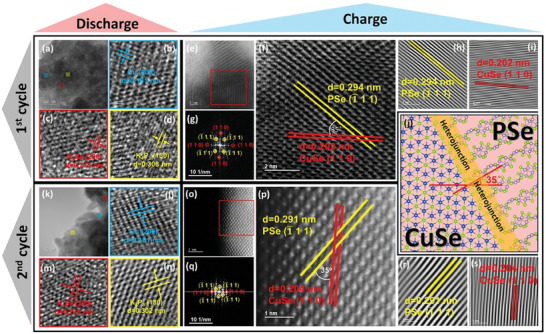
Ex situ TEM characterizations. a–d) HRTEM images of Cu_3_PSe_4_ electrode after being discharged to 0.01 V of the 1^st^ cycle. e,f) HAADF‐STEM images, g) FFT pattern, and h,i) IFFT images after being charged to 3 V of the 1^st^ cycle. j) schematic diagram of the heterohunction. k–n) HRTEM images of Cu_3_PSe_4_ electrode after being discharged to 0.01 V of the 2^nd^ cycle. e,f) HAADF‐STEM images, g) FFT pattern, and h–i) IFFT images after being charged to 3 V of the 2^nd^ cycle.

Based on the above experiments and theoretical calculations, we explored the K^+^ storage mechanism of Cu_3_PSe_4_ through the ex situ HRTEM and ex situ high angle annular dark field scanning transmission electron microscopy (HAADF‐STEM) images, respectively. From the lattice fringes of **Figure** **3**a–d, the products after the first fully discharged are cubic Cu (blue area), cubic K_2_Se (red area), and orthorhombic K_4_P_3_ (yellow area). The d‐spacings of 0.181, 0.275, and 0.303 nm correspond to the crystal planes of Cu (200), K_2_Se (220), and K_4_P_3_ (130), respectively. The Cu_3_PSe_4_ forms the above products through the conversion reaction of multiple electron transfers in the first discharge process. The HAADF‐STEM image shows (Figure 3e), the CuSe/PSe heterojunction formed after being fully charged (Figure 3f). Figure 3g is the image after Fast Fourier Transform (FFT), where the diffraction spots correspond to the crystal planes of PSe ($\bar{1}$11) and CuSe (110). Through the inverse Fast Fourier Transform (IFFT) images, the obtained d‐spacings are 0.294 and 0.202 nm respectively (Figure 3h,i). Furthermore, when PSe ($\bar{1}$11) and CuSe (110) grow parallel to each other, resulting a large lattice mismatch in 31.3% [(0.294‐0.202)/0.294 = 31.3%]. Therefore, the growing angle between PSe ($\bar{1}$11) and CuSe (110) plane must be grown in a tilted manner (e.g., 35°), then the lattice mismatch is greatly reduced to 15.9%, as shown in Equation (7).^[^ ^57^ ^]^

(7) $$\begin{array}{c} lattice\;misfit\;\left(\delta \right)\;=\frac{\left|dPSe\left(\bar{1}11\right)-dCuSe\left(110\right)/cos\left(35^{\circ }\right)\right|}{dPSe\left(\bar{1}11\right)}=15.9\% \end{array}$$

Here, a CuSe/PSe heterojunction is in situ constructed after the first potassiation/depotassiation from Cu_3_PSe_4_. We further checked the condition of the electrode material after the second cycle. Similarly, the CuSe/PSe heterojunction also has the same products after fully discharged (Figure 3k–n), cubic Cu (blue region), cubic K_2_Se (red region), and orthorhombic K_4_P_3_ (yellow region). The d‐spacings of 0.181, 0.278, and 0.302 nm correspond to the crystal planes of Cu (200), K_2_Se (220), and K_4_P_3_ (130), respectively. The HAADF‐STEM images also show (Figure 3o–p), the products of the CuSe/PSe heterojunction after being charged still correspond to the heterojunction composed of PSe and CuSe, pointing out its high reversibility of the heterojunction. The diffraction spots of the image after FFT also correspond to the crystal planes of PSe ($\bar{1}$11) and CuSe (110) (Figure 3q). Through the image of IFFT, the d‐spacings are 0.291 and 0.204 nm (Figure 3r,s), respectively. In particular, CuSe/PSe p‐n heterojunction structure after long‐term cycling is validated by ex situ XRD and HRTEM, exhibiting that high reversibility in PIB (Figures S6 and S7, Supporting Information). At the same time, the growing angle between them is also 35°, reducing the lattice mismatch to14.4%. The CuSe/PSe heterojunction is formed irreversibly by ternary Cu_3_PSe_4_ in the first stage, and the heterojunction is highly reversible in subsequent cycles, which can induce spontaneous electron transfer and accelerate ion/electron transfer, as shown in Figure 3j. Here we also propose the potassium storage mechanism (Equations (8)–(10)):

First Cycle:

(8) $$\begin{array}{c} Discharge:\;Cu_{3}PSe_{4}+\frac{28}{3}K^{+}+\frac{28}{3}e^{-}\to 4K_{2}Se+\frac{1}{3}K_{4}P_{3}+3Cu \end{array}$$

(9) $$\begin{array}{c} Charge:\;4K_{2}Se+\frac{1}{3}K_{4}P_{3}+3Cu\to 3CuSe+PSe+\frac{28}{3}K^{+}+\frac{28}{3}e^{-} \end{array}$$

The following cycles:

(10) $$\;3CuSe+PSe+\frac{28}{3}K^{+}+\frac{28}{3}e^{-}↔4K_{2}Se+\frac{1}{3}K_{4}P_{3}+3Cu$$

### 3.2 Heterojunction Characterizations of CPS‐h and Its High Reversibility

**Figure 4:**
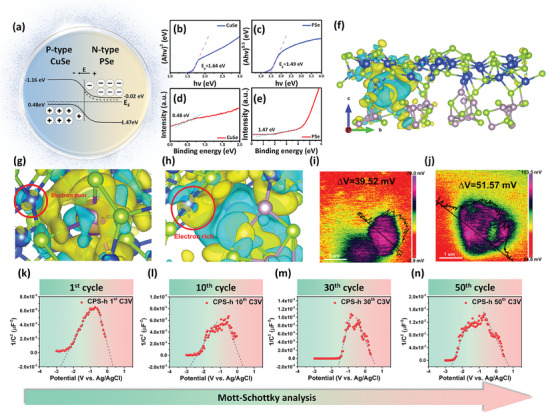
Heterojunction characterization. a) Band diagram for p‐type CuSe and n‐type PSe. b,c) Tauc's bandgap plots, d,e) UPS valence band spectra of CuSe and PSe. f) The charge density difference diagram of CPS‐h with K^+^ adsorbed. The charge density difference of g) CuSe and h) CPS‐h with K^+^ adsorbed. The KPFM results of i) Cu_3_PSe_4_ and j) CPS‐h. The Mott‐Schottky plot of CPS‐h at the k) 1^st^, l) 10^th^, m) 30^th^, and n) 50^th^ cycle.

After exploring the potassium storage mechanism, Cu_3_PSe_4_ was electrochemically constructed in situ into a heterostructure of CuSe and PSe (denoted as CPS‐h). Since we can't directly measure the distinct semiconductor properties of the in situ formed CuSe and PSe due to their inseparability within the electrode, we separately synthesized and identified the energy gaps and positions of CuSe (PDF 01‐086‐1240) and PSe (PDF 04‐007‐1805) powders. The XRD patterns for CuSe and PSe, shown in Figures S8 and S9 (Supporting Information). The schematic of the energy bands and the developed heterojunction model, as illustrated in **Figures** **4**a and S10 (Supporting Information), demonstrate bandgaps of 1.64 eV for CuSe (a direct semiconductor) and 1.49 eV for PSe (an indirect semiconductor), respectively.^[^ ^58^, ^59^ ^]^ These values were determined using the Tauc method, as shown in Figure 4b,c. To further classify the semiconductor types and the p‐n heterojunction characteristics, Ultraviolet Photoelectron Spectroscopy (UPS) was employed to examine the electronic band structures of CuSe and PSe. Figure 4d,e shows the valence band maximum (VBM) edges for CuSe and PSe at 0.48 and 1.47 eV, respectively. Given the differential positioning of the Fermi levels, spontaneous electron transfer is prompted within the CuSe/PSe heterostructure when potassium ions are introduced; holes from the p‐type CuSe migrate toward the n‐type PSe, while electrons travel in the opposite direction, from n‐type to p‐type. As carriers cross the interface and recombine, equilibrium is achieved, generating a depletion region of positively and negatively charged layers. This interfacial charge redistribution and the formation of an electric field between the depletion layers can effectively adjust the local structure of the material, particularly its electronic structure.^[^ ^60^ ^]^ Energy levels in n‐type and p‐type semiconductors adjust to align. At the tightly bonded interface, band edge bending occurs to achieve thermodynamic equilibrium.^[^ ^61^ ^]^ As a result, electrons and K ions move directly between CuSe and PSe, optimizing the distribution of charged entities.^[^ ^25^ ^]^ Ex situ XPS technique is employed for identifying electrode states at various potentials (OCV, D0.01 V, C3V). In the Cu 2p energy spectrum (Figure S11, Supporting Information), binding energies for Cu 2p_1/2_ and Cu 2p_3/2_ shift to lower values during discharge (952.60 and 932.80 eV to 951.92 and 932.08 eV, respectively), indicating a change from Cu^+^ to Cu^0^ after complete potassiation. Upon charging to 3 V, the binding energies are 952.47 and 932.65 eV, respectively. Compared to pure CuSe spectrum, where binding energies are 952.50 and 932.68 eV, the formation of a heterojunction results in spontaneous electron transfer from n‐type PSe to p‐type CuSe. This increases the shielding effect on Cu, thereby lowering the Cu 2p binding energy at C3V. In addition, a binding energy shift is seen in P 2p (Figure S12, Supporting Information). The binding energy of P 2p_1/2_ and P 2p_3/2_ shifts toward lower values (from 133.72 and 131.46 eV to 133.11 and 131.26 eV, respectively) when discharged to 0.01 V. This suggests that following the formation of K_4_P_3_, the strong electron‐pushing capability of K increases shielding effect of P, thereby reducing binding energy.^[^ ^62^ ^]^ Charging to 3 V creates a CuSe/PSe heterojunction, with P 2p binding energy surpassing pure PSe. At C3V, values are 133.30 and 130.36 eV versus 133.25 and 131.42 eV. With a 3:1 CuSe to PSe ratio, significant electron transfer from PSe to CuSe occurs, diminishing P's shielding and raising its binding energy. These ex situ XPS results further corroborate the spontaneous electron transfer prompted by the built‐in electric field. Additionally, we have constructed a differential charge density diagram depicting potassium ions adsorbed in the CuSe unit cell, the PSe unit cell and the interface of the CPS‐h heterojunction (Figure 4f–h; Figures S13 and S14, Supporting Information). Upon potassium ion adsorption in the CuSe unit cell, Cu atoms demonstrate charge depletion characteristics (Figure 4g). Following heterojunction formation, n‐type PSe diffuses electrons to p‐type CuSe, harmonizing Fermi energy levels. At the CPS‐h heterojunction, Cu atoms notably exhibit charge accumulation characteristics after potassium ion adsorption (Figure 4h). The formation of the heterojunction appears to boost bidirectional charge transfer, quicken interface electron transport, and amplify ion diffusion kinetics, which in turn supports ion storage capacity in the K^+^ electrode.^[^ ^63^ ^]^

The Kelvin probe force microscopy (KPFM) technique was employed to confirm the formation of the built‐in electric field by measuring the surface charge density. At the same distance and tip height, the surface potentials for Cu_3_PSe_4_ powder and CPS‐h were approximately 39.52 and 51.57 mV respectively (Figure 4i,j). The correlation between the surface potential and the built‐in electric field (BIEF) can be depicted by Equation (11) as per KPFM measurements:^[^ ^64^ ^]^

(11) $$E\;={\left(-\frac{2Vs\rho }{\varepsilon \varepsilon_{0}}\right)}^{\frac{1}{2}}\;$$

A potential increase of 12.05 mV indicates a robust built‐in electric field at the CuSe/PSe heterojunction. Here, E represents the value of BIEF, Vs is the surface potential, ρ indicates surface charge density (C m^−2^), ε refers to the low‐frequency dielectric constant, and ε0 is the vacuum dielectric permittivity (F m^−1^). According to Mott‐Schottky theory, the space charge capacitances of n‐type and p‐type semiconductors are defined by Equations (12) and (13), assuming the capacitance of the Helmholtz layer can be disregarded.^[^ ^65^ ^]^

(12) $$\begin{array}{c} \frac{1}{C^{2}}=\frac{2}{\varepsilon \varepsilon_{0}eN_{d}}\;\left(E-E_{FB}-\frac{kT}{e}\right)\;for\;n-type\;semiconductor \end{array}$$

(13) $$\begin{array}{c} \frac{1}{C^{2}}=\;-\frac{2}{\varepsilon \varepsilon_{0}eN_{a}}\left(E-E_{FB}-\frac{kT}{e}\right)\;for\;p-type\;semiconductor \end{array}$$

In the equations, C represents the depletion‐layer capacitance per unit surface area, N_d_ and N_a_ are donor and acceptor densities, respectively. ε_0_ is the vacuum permittivity, ε is the dielectric constant of the semiconductor, E is the electrode potential, E_FB_ stands for the flat‐band potential, and kT∕e is a temperature‐dependent term in the Mott‐Schottky equation. The nature of a semiconductor can be determined from the slope in the Mott‐Schottky diagram; positive indicates n‐type, and negative suggests p‐type. The negative slope of the Cu_3_PSe_4_ powder in Figure 1c indicates its p‐type semiconductor nature. Post electrochemical reaction with potassium ions, a slope reversal is observed in the Mott‐Schottky plot (Figure 4k), evidenced by an inverted “V” pattern, confirming successful construction of an in situ p‐n junction with a built‐in electric field.^[^ ^66^ ^]^ CPS‐h was also measured after the 10^th^, 30^th^, and 50^th^ cycle (Figure 4l–n), with results maintaining the inverted “V” pattern, suggesting the highly reversible CuSe/PSe heterostructure offers stable performance for ion/electron transport.

### 3.3 The Electrochemical Performances of CPS‐h/G

**Figure 5:**
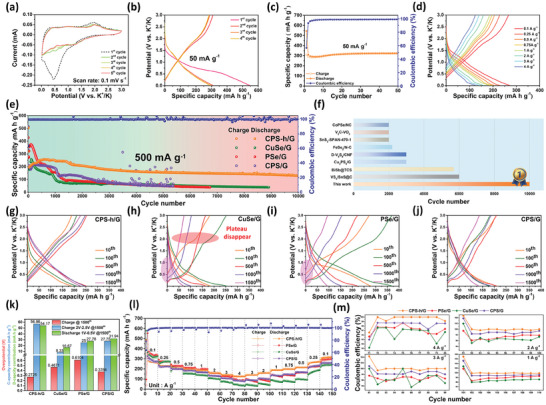
Electrochemical performance of CPS‐h/G. a) CV curves. b) GCD curves at 50 mA g^−1^ and corresponding c) cycling performance. d) GCD curves at different current densities. e) Long‐term cycling performance. f) Cycle number comparison at the field of K^+^ storage. g) The GCD curves at different cycles of (g) CPS‐h/G, h) CuSe/G, i) PSe/G, and j) CPS/G. k) The charge overpotential and capacity contribution diagram of four electrodes at the 1500^th^ cycle. j) Rate capability of four electrodes. m) Columbic efficiency at different current densities of four electrodes.

CPS‐h, an in situ electrochemically constructed heterojunction with a reversible P‐N heterostructure, benefiting from the inherent electric field at the heterointerface to boost ion/electron conduction, delivering superior electrochemical performance as validated using CPS‐h/G electrodes in CR2032 coin. During the initial discharge, a reduction peak appeared at 1.1 V, suggesting potassium ion movement into the Cu_3_PSe_4_ crystal, leading to its conversion into K_2_Se and K_4_P_3_. Subsequently, a reduction peak at 0.44 V marked the formation of an irreversible SEI layer. On the contrary, during the first charge, a broad peak at 0.3 V signifies depotassiation in the graphite buffer, followed by oxidation peaks at 0.96 and 1.96 V, due to K_4_P_3_ and K_2_Se depotassiation, resulting in CuSe/PSe (**Figure** **5**a). Figure 5b shows the galvanostatic charge‐discharge curve at 50 mA g^−1^ current density, with strong overlap after the second cycle, reflecting the high reversibility of CPS‐h/G electrode.

Figure 5c illustrates the cycle performance, maintaining a highly reversible capacity of 321.25 mAh g^−1^ after 50 cycles. Figure 5d depicts the galvanostatic charge–discharge (GCD) curves of CPS‐h/G electrode at varying current densities. With current densities of 0.1, 0.25, 0.5, 0.75, 1, 2, 3, and 4 A g^−1^, the electrode shows reversible discharge capacities of 271.6, 230.4, 207.5, 196.5, 186.4, 160.0, 141.7, and 125.6 mAh g^−1^, respectively. Higher current densities increase charge/discharge overpotential, while the reaction platform is retained, confirming high stability of the electrode.^[^ ^67^ ^]^ Long‐term cycling performance of four electrodes at 500 mA g^−1^ is presented in Figure 5e. CuSe/G, PSe/G, and CuSe/PSe/G (CPS/G) display capacity fade in early cycles, suggesting instability. PSe/G shows severe capacity fade due to volume expansion. CPS/G, supplemented with P, exhibits stable initial cycling performance, implying effective Se loss suppression via the P−Se bond. In contrast to CPS/G, which lacks heterostructure and undergoes severe capacity fade after 1000 cycles, the CPS‐h/G electrode, with its robust p‐n heterostructure, displays excellent mechanical strength and prevents electrode material loss. Over long‐term cycling, only the CPS‐h/G electrode sustains over 10000 cycles, maintaining a 130 mAh g^−1^ capacity (continuous operation for 6974 h, capacity loss rate of 0.005% per cycle). Figure 5f presents a comparative analysis of the half‐cell cycle durations in the potassium‐ion battery system. Notably, CPS‐h/G achieved a remarkable 10000 cycles, outperforming many PIBs.^[^ ^31^, ^68^, ^69^, ^70^, ^71^, ^72^, ^73^, ^74^ ^]^ Further, the GCD curves for the 10^th^, 100^th^, 500^th^, 1000^th^, and 1500^th^ cycles (Figure 5g–j) revealed the deteriorating reversibility of the CuSe/G electrode; the Se charging plateau disappeared after 500 cycles, suggesting progressive loss or deactivation of Se during cycling (Figure 5h).

Figure 5i,j indicates PSe/G and CPS/G electrodes maintain the Se plateau beyond 1500 cycles due to the robust P−Se chemical bond reducing the Se shuttling effect. Yet, these along with CuSe/G, experience capacity drops post 1000 cycles, likely from high overpotentials leading to incomplete reactions. In contrast, the CPS‐h/G electrode not only retains the Se plateau over extensive cycling but also demonstrates the least charging overpotential among the four electrodes (Figure 5g). As depicted in Figure 5k, the charging overpotential of CPS‐h/G after 1500 cycles is just 0.2725 V, significantly lower than those of CuSe/G (0.4671 V), PSe/G (0.6104 V), and CPS/G (0.3766 V). Moreover, at the 1500^th^ cycle, within the discharge range of 1 to 0.5 V, CPS‐h/G, CuSe/G, PSe/G, and CPS/G contributed capacities of 54.17, 16.67, 27.78, and 31.94 mAh g^−1^ respectively. Similarly, within the charge range of 2 to 2.5 V, they contributed capacities of 56.96, 8.33, 25, and 27.78 mAh g^−1^ respectively. Thus, in both conversion reactions' voltage ranges, CPS‐h/G electrode consistently provided the highest capacity, affirming its superior electrochemical performance. In rate performance tests of the four materials (Figure 5l), CPS‐h/G electrode was the most stable and achieved a reversible rate of 125.6 mAh g^−1^ at 4 A g^−1^ capacity. As current density returned from 4 to 1 A g^−1^, other three electrodes exhibited instability. Coulombic efficiency was examined in this interval (Figure 5m): P‐containing electrodes maintained relative stability, while CuSe/G showed worst stability and Coulombic efficiency. Among the four, CPS‐h/G electrode had the highest Coulombic efficiency at nearly 100%.

### 3.4 The Potassium Ion Kinetics Analysis

**Figure 6:**
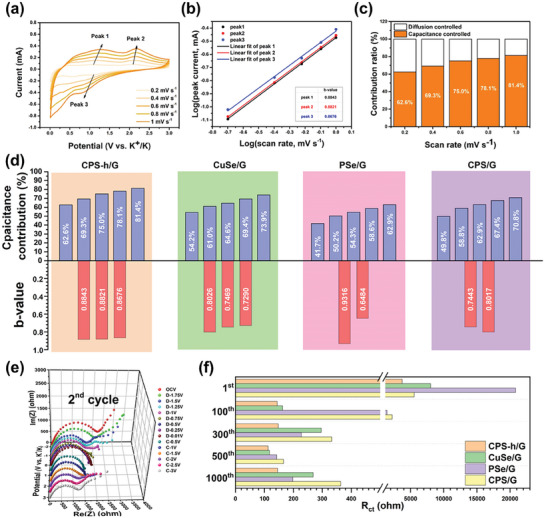
Kinetic analysis. a) CV curves at various scan rates of CPS‐h. b) Linear fitting profiles of log (i, peak current) versus log (v, scan rate) of CPS‐h. c) Capacitance contribution of CPS‐h. d) The statistic diagram of capacitance contribution and b‐value for four electrodes. e) The in situ EIS analysis after the formation of CPS‐h. f) The charge transfer resistance of four electrodes at the 1^st^, 100^th^, 300^th^, 500^th^, and 1000^th^ cycle.

CPS‐h/G demonstrates superior K^+^ storage and long‐term cycling stability, correlated with its high‐rate pseudocapacitive behavior. To examine the benefits of the heterointerface in the PIB system and its dynamic response, we measured and calculated pseudocapacitive contributions using cyclic voltammetry (CV) at various scan rates (0.1–1.0 mV s^−1^). **Figure** **6**a presents an analysis of capacitive effect on CPS‐h/G at these rates, with increased similarity in shape as scan rate grows, suggesting its outstanding electrochemical stability.^[^ ^75^ ^]^ The relationship between peak current (i) and scan rate (v), described by Equation (14), indicates capacitive effect: a b‐value of 0.5 reveals a diffusion‐controlled process, while a b‐value of 1.0 implies a capacitive‐controlled process. Plotting log(i) against log(ν) helps calculate the b‐value. Peaks 1 to 3 show b‐values of 0.88, 0.88, and 0.86 respectively (Figure 6b). The capacitive contribution is quantified using Equation (15).^[^ ^76^ ^]^

(14) $$i\;=\;av^{b}\;,\;\log\left(i\right)=\;blog\left(v\right)+\log\left(a\right)$$

(15) $$i\;\left(v\right)=k_{1}\;v+k_{2}v^{\frac{1}{2}}$$

where k_1_ and k_2_ are constant values corresponding to capacitance‐controlled and diffusion‐controlled processes, respectively. Figure 6c illustrates pseudocapacitive contributions at varying scan rates, with capacitance ratios being 62.6%, 69.3%, 75.0%, 78.1%, and 81.4% at scan rates of 0.2, 0.4, 0.6, 0.8, and 1.0 mV s^−1^, respectively. Additionally, kinetic responses of CuSe/G, PSe/G, and CPS/G electrodes were analyzed (refer Figures S15–S17, Supporting Information), compiled in Figure 6d. Among them, CuSe/G exhibits higher pseudocapacitive contribution, yielding capacitance ratios of 54.2%, 61.0%, 64.6%, 69.4%, and 73.9% at corresponding scan rates. In contrast, PSe/G contributes the least, with capacitance ratios of 41.7%, 50.2%, 54.3%, 58.6%, and 62.9% respectively. Capacitance ratios for CPS/G electrodes fall between the previous two, at 49.8%, 58.8%, 62.9%, 67.4%, and 70.8%. Following a linear fit, we calculated the b‐values for each electrode: CuSe/G, PSe/G, and CPS/G averaged at 0.7595, 0.79, and 0.773, respectively. CPS‐h/G surpasses the others in both pseudocapacitive contribution at each scan rate and the average b value (0.878). This illustrates how the built‐in electric field and lattice distortion at the interface can enhance electrode kinetics, resulting in superior rate capability and long‐term stability.

Using the Galvanostatic Intermittent Titration Technique (GITT) with pulsed currents of 0.1 A g^−1^ for 0.33 h (and a 1 h rest interval), we determined the K^+^ diffusion coefficient in CPS‐h/G, CuSe/G, PSe/G, and CPS electrodes. Before GITT tests, cells underwent three discharge/charge cycles at 0.05 mA g^−1^. Fick's second law allows calculation of D_K+_ from the GITT curve using a simplified equation (Equation 16):^[^ ^77^ ^]^

(16) $$D_{K+}=\frac{4}{\pi \tau }\;L^{2}{\left(\frac{▵E_{S}}{▵E_{\tau }}\right)}^{2}$$

where τ, ΔE_s_, ΔE_τ_, and L represent the current pulse time, the constant voltage change induced by the current pulse, the potential change in the steady‐state current pulse, and the electrode thickness, respectively. During potassiation/depotassiation, the diffusion coefficients of the four electrodes are displayed in Figure S18 (Supporting Information). CPS‐h/G exhibited the highest average diffusion coefficient (Avg. D_K+_ = 2.70 × 10^−10^), compared to CuSe/G, PSe/G, CPS/G which were 2.34 × 10^−10^, 1.82 × 10^−10^, and 2.68 × 10^−10^, respectively. And, the D_K+_ values of CPS‐h/G are at an above the average level in the other heterojunction systems (Table S1, Supporting Information). Additionally, we measured the interfacial transfer resistance (R_ct_) of the CPS‐h/G electrode in the second cycle using an in situ electrochemical impedance spectroscopy (EIS) technique, after heterojunction formation. As seen in Figure 6e, the interfacial transfer impedance remained stable throughout the discharge/charge process, showing improvement from the high interfacial transfer impedance (3737 ohm) of CPS‐h/G in the first cycle (Figure 6f). Thanks to the in situ engineering of the heterojunction interface, the heterojunction formation effectively enhances bilateral charge transfer, accelerates interfacial electron transport, facilitates ion diffusion kinetics, and provides sustainable ion storage capacity in K^+^ electrodes. We also measured the interfacial transfer impedance of each electrode in the 1^st^, 100^th^, 300^th^, 500^th^, and 1000^th^ cycles, as illustrated in Figure 6f. In the first cycle, the R_ct_ of CPS‐h/G, CuSe/G, PSe/G, and CPS/G were 3737, 8035, 20 826, 5552 ohm, respectively, with the high resistance of PSe likely due to inherently poor conductivity. However, after 100 cycles, the interfacial transfer impedance of the four significantly decreased, owing to the SEI layer produced and electrochemical reconstruction, and remained stable in subsequent cycles.^[^ ^78^ ^]^ CPS‐h/G consistently exhibited the lowest interfacial transfer resistance after each cycle, underscoring its superior charge transfer performance.

### 3.5 DFT Computations of the CPS‐h Heterojunction

**Figure 7:**
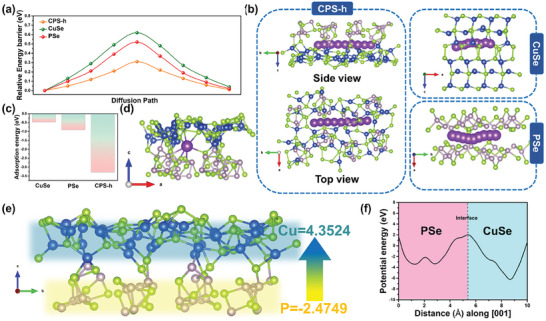
DFT calculations. a) The K^+^ diffusion barrier diagram and the corresponding b) diffusion path. c) The K^+^ adsorption energy of CPS‐h, CuSe, and PSe. d) The structure model of CPS‐h with K^+^ adsorbed. e) Bader charge analysis of CPS‐h. f) The Plane‐averaged electrostatic potential of CPS‐h.

Through DFT calculations, we examined the heterointerface's impact on reaction kinetics, focusing on K^+^ storage adsorption energy and the intralattice diffusion barrier, as shown in **Figure** **7**a. Energy barriers, representing the energy for alkali metal transport in CPS‐h, CuSe, and PSe electrodes, were also evaluated. Lower energy barriers suggest faster transport and storage of alkali metal atoms, hence improved electrochemical performance.^[^ ^79^ ^]^ Moreover, lattice distortion at the heterogeneous interface enables it to act as a rapid shuttle channel for potassium ions, giving CPS‐h the lowest diffusion energy barrier across all migration sites.^[^ ^80^ ^]^ The energy barrier of PSe is intermediate, with its 2D layered structure offering a lower diffusion barrier than CuSe. The diffusion paths of the three materials are depicted in Figures 7b and S19–S21 (Supporting Information), and these paths conform to Fick's first law as described in Equation (17).

(17) $$J\;=\;-D\frac{dC}{dx}$$

where J is the diffusion flux, D is the diffusion coefficient, and dC/dx is the concentration gradient. The elevated diffusion coefficient of the heterojunction enhances diffusion flux, thus offering superior ion diffusion kinetics compared to pure materials.^[^ ^23^ ^]^ During electrochemical reactions, potassium ions initially adsorb onto the material surface. Having efficient adsorption sites can expedite this reaction. Based on DFT calculations and depicted in Figures 7c,d and S22–S24 (Supporting Information), CPS‐h demonstrated a significantly higher potassium ion adsorption energy (−3.30 eV) than both CuSe (−0.47 eV) and PSe (−0.91 eV). This indicates that the multicomponent heterostructure of CPS‐h strongly adsorbs potassium ions, which can further speed up the potassiation process.^[^ ^69^ ^]^ To further understand the superior charge transport capability of the heterojunction, we conducted Bader charge analysis, depicted in Figure 7e. The charge of each element in CPS‐h is detailed in Table S2 (Supporting Information). The Bader charges of Cu and P are 4.3524 and −2.4749, respectively, suggesting that upon heterojunction formation, electrons are transferred from n‐type PSe to p‐type CuSe, in agreement with earlier charge density difference map results. This additional electric field could facilitate swift electron transport within the electrode during K^+^ insertion/extraction, thereby enhancing charge transfer kinetics. Additionally, the inherent electric field reduces activation energy and accelerates charge transport, enhancing charge separation during charge/discharge. Compared to pure materials, the heterostructure transfers more electrons to its surface, absorbing more alkali metal ions for charge balance. Figure 7f depicts the calculated electrostatic potential of the CPS‐h heterostructure along the normal [0 0 1] direction, providing insight into its internal potential. The average potential on the CuSe side is lower than on the PSe side, as it descends along the [0 0 1] direction of the heterostructure. This discrepancy causes charges to move from PSe to CuSe, emphasizing how the built‐in electric field of CPS‐h optimizes electron transport and potassium storage kinetics.^[^ ^81^ ^]^ Additionally, the thermal stability of CPS‐h was investigated by ab initio molecular dynamics (AIMD) simulations, using a time step of 2 fs for a total of 10 ps (Figure S25, Supporting Information). It can be observed that the energy fluctuation can be kept within a very small energy window at 300 and 500 K, and the heterointerface remains intact despite slight distortions, indicating that the CPS‐h heterostructure can exist stably at 300 and 500 K.

### 3.6 The Applications on Potassium Ion Full Batteries and Hybrid Capacitors

**Figure 8:**
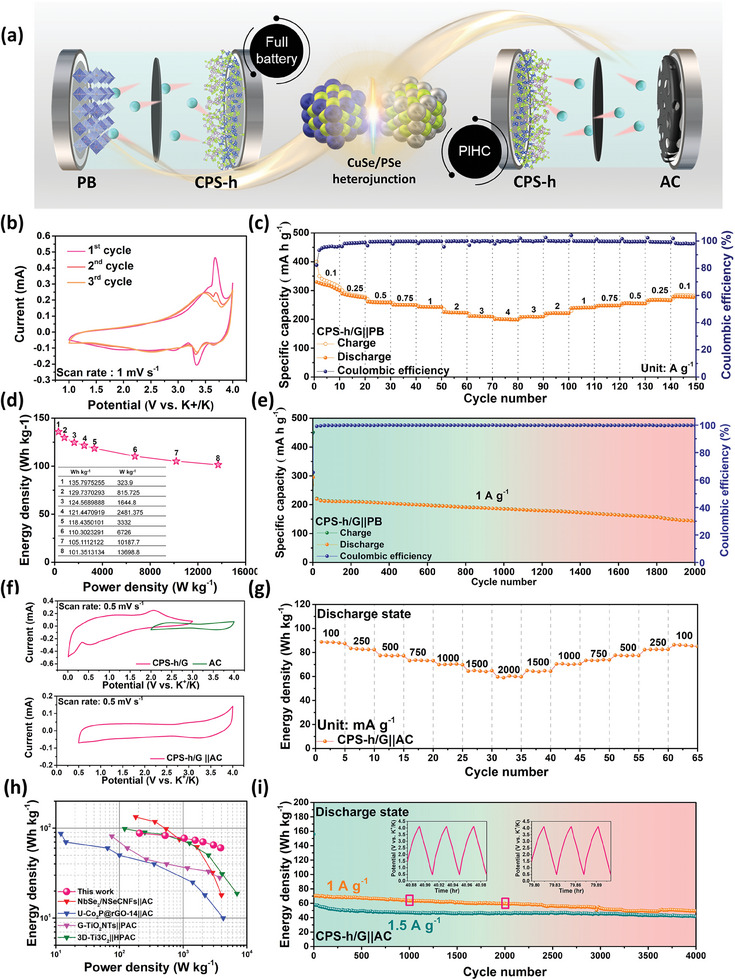
Potassium ion full batteries and PIHCs of CPS‐h/G||PB. a) Schematic illustration of CPS‐h/G ||PB full battery and PIHC. b) The CV profiles at a scan rate of 1 mV s^−1^. c) Rate capability at different current densities ranging from 0.1 to 4 A g^−1^ and d) the corresponding energy densities and power densities in Ragone plot. e) Long‐term cycling performance at 1 A g^−1^. f) CV curves of a half cell and a full cell. g) Rate performance at the current densities ranging from 100 to 2000 mA g^−1^. h) Ragone plot of the CPS‐h/G||AC PIHC full cell in comparison with the reported PIHCs. i) The long‐term cycling performance at 1 and 1.5 A g^−1^. The insets present the voltage profiles after 1000 and 2000 cycles.

We evaluated the practicality of CPS‐h/G anode by assembling a full potassium‐ion battery with Prussian blue (PB) as the cathode and CPS‐h/G as the anode, resulting in a CPS‐h/G||PB battery, as shown in **Figure** **8**a. The XRD pattern and half‐cell performance of PB are shown in supplementary figure (Figures S26–S29, Supporting Information). From the CV curves at a scan rate of 1 mV s^−1^ (Figure 8b), the overlapping peaks highlight the high reversibility of the chemical reaction and the suitable voltage of 1.0–3.8 V for the CPS‐h/G||PB full cell window. Normalized charge‐discharge curves of the CPS‐h/G half‐cell, PB half‐cell, and CPS‐h/G||PB full‐cell are presented in Figure S30 (Supporting Information). The rate performance of CPS‐h/G||PB full cells was measured (Figure 8c; Figure S31, Supporting Information). Discharge capacities were 278, 267, 255, 247, 240, 221, 209, 200 mAh g^−1^, respectively. Despite high current density cycling at 4 A g^−1^, the CPS‐h/G||PB full battery maintained excellent stability and over 98% Coulombic efficiency. With its high charge–discharge platform, the full cell attains an energy density of 135.79 Wh kg^−1^ and a power density of 13 698.8 W kg^−1^, considering both electrodes' activity (Figure 8d). Standard methods were employed for battery system calculations. The long‐term cycle test (Figure 8e), based on the anode active material's total weight, demonstrates that the CPS‐h/G||PB PIB full battery achieves over 2000 cycles at 1 A g^−1^, offering a reversible capacity of 143.12 mAh g^−1^. There's a 0.018% capacity fade each cycle. Impressively, one CPS‐h/G||PB button‐type battery powered 71 red LED bulbs (inset of Figure 8e).

Benefiting from the robust kinetics and stability of CPS‐h/G, we developed potassium‐ion hybrid capacitors (PIHC) using activated carbon (AC) cathodes and CPS‐h/G anodes. The superior electron and K ion transport of the heterostructure is exemplified in the CPS‐h/G||AC configuration (Figure 8f). During charging, cations transform with the anode, and anions adsorb to the cathode. Due to aluminum foil attrition in FIS‐based electrolytes above 4.0 V, we set the AC half‐cell voltage window from 2.0 to 4.0 V.^[^ ^82^ ^]^ Figures S32–S35 (Supporting Information) depicts AC half‐cell performance and CV curve, exhibiting a consistent specific capacity of 37.5 mAh g^−1^ over 1000 cycles at 1 A g^−1^. At a higher current density of 4 A g^−1^, it retains a specific capacity of 30 mAh g^−1^. Figure 8g depicts the redox peaks of half and full cells. The CV curves of PIHCs display quasi‐rectangular shapes with no polarization, functioning stably at 0.5–4 V operating voltage. A slight shape deviation indicates both Faradaic and non‐Faraday reactions, thereby enhancing energy (E) and power density (P) when considering the integral and conversion formula.

(18) $$E\;=\;I\int_{t_{2}}^{t_{1}}V\left(t\right)dt/m\;=\;P\times t$$

where I and t are the constant current and period of the discharge process, and m is the total mass loading of the anode and cathode. For optimal power output, we selected a 1:1 cathode to anode mass ratio.^[^ ^83^ ^]^ Figure S36 (Supporting Information) presents the GCD curve, indicating a consistent platform shift aligned with individual cathode and anode behaviors. Figure S37 (Supporting Information) reveals GCD curves at different rates, underscoring the CPS‐h/G||AC system's excellent rate performance, as seen by the minimal IR drop at elevated current densities. Figure 8h illustrates the CPS‐h/G||AC full capacitors achieving energy densities from 88.42 to 60.32 Wh kg^−1^ at currents ranging from 0.1−2 A g^−1^, based on the total active mass of CPS‐h/G and AC. The Ragone diagram, plotting specific energy against specific power on logarithmic axes, enables comparison across different systems (Figure 8i).^[^ ^84^, ^85^, ^86^, ^87^ ^]^ CPS‐h/G||AC exhibits superior electrochemical performance at current densities of 1 and 1.5 A g^−1^, it consistently delivers energy densities of 49.7 and 41.6 Wh kg^−1^ across 4000 cycles. Both measurements have energy retention ≈70%, and voltage distributions at the 1000^th^ and 2000^th^ cycles remain stable without pronounced overpotential, as illustrated in inset of Figure 8i. It was noteworthy that CPS‐h/G demonstrates cycle stability for 2000 and 4000 cycles in full cells and hybrid capacitors, respectively (Table S3, Supporting Information).

## 4 Conclusion

We report an electrochemical in situ method to create the highly reversible p‐n heterostructure of CPS‐h that retains the P−P bond in K_4_P_3_ and realizes the thermodynamically favorable coexistence of CuSe and PSe. Compared with the previous heterostructure materials, CPS‐h benefits include: 1) In situ electrochemical conversion from Cu_3_PSe_4_ to p‐n heterojunction eliminates the need for costly and complicated processes like drying, separation, and high‐temperature manufacturing. 2) Electrochemical processes create large‐scale heterointerfaces with the nanoscale dimensions necessary for energy storage. 3) The reaction follows the minimum free energy principle, ensuring a stable heterostructure. At the process level, the heterojunction of traditional multi‐step synthesis is replaced by in situ electrochemical induction, and the nanocrystal effect is directly realized by molecular‐level cracking engineering. In terms of electrochemical performance, the introduction of P−Se bonds suppresses the Se shuttle effect and the inherent built‐in electric field achieves ultra‐stable cycles, even applied to potassium‐ion full batteries and hybrid capacitors. The ion/electron transport is accelerated by the space‐charge region and lattice mismatch excited by the abundant molecular contact interface. This means the p‐n heterojunction remains consistent during subsequent electrochemical reactions with potassium ions, without concerns of phase transitions. This study transforms the traditional, labor‐intensive method of producing heterojunctions and offers enhanced convenience and practicality for the in situ production of p‐n heterostructures for potassium‐ion storage systems. Moreover, its applicability may go beyond potassium ions, hinting at a broader potential for various ion energy storage systems.

## 5 Experimental Section

### Preparation of Cu_3_P

Cu_3_P was synthesized via a facile and largely scalable high energy ball milling method under the protection of Ar. Briefly, 0.96 g of commercial copper powder, 0.155 g of red phosphorus. After sealing in an Argon filled glovebox, the mixture was ball‐milled at 400 rpm for 30 h.^[^ ^88^ ^]^

### Preparation of Cu_3_PSe_4_

The Cu_3_PSe_4_ was synthesized by a simple ball milling method. First, the molar ratio of as‐synthesized Cu_3_P and Se powder were sealed under Ar atmosphere before ball milling at 400 rpm 24 h. The Cu_3_PSe_4_/G was synthesis via a ball milling method with Cu_3_PSe_4_ an graphite (2:1) at 400 rpm for 24 h.

### Preparation of CuSe

The synthesis of CuSe was carried out using a facile one‐pot solution strategy with Ethylene glycol as a solvent. CuCl (1.0 mmol), SeO_2_ (1.0 mmol) were dissolved into 20 mL EG with magnetic stirring to form the precursor solution at room temperature under Ar atmosphere for 1 h. The obtained solution was then transformed into a three‐neck bottle followed by the addition of 1.0 mL N_2_H_4_·H_2_O and kept magnetically stirring for ≈30 min. After that, the reaction solution was heated to 160 °C and kept stirring for 1 h to generate a dark‐brown mixture. After naturally cooling down to room temperature, the final products were collected by centrifugation and washed with DI water and ethanol several times.^[^ ^89^ ^]^ The CuSe/G was synthesis via a ball milling method with CuSe an graphite (2:1) at 200 rpm for 24 h.

### Preparation of PSe

First, 3.2 mmol of P and Se powder were sealed in a vacuum quartz tube, heating at 310 °C for 8 h with a rate of 3 °C min^−1^.^[^ ^90^ ^]^ The PSe/G was synthesis via a ball milling method with PSe an graphite (2:1) at 200 rpm for 24 h.

### Preparation of CuSe/PSe/G

The as‐synthesized CuSe and PSe powder were combined with molar ratio in 3:1. Then add the above mixture and graphite (2:1) and ball mill at 200 rpm for 24 h.

### Preparation of PB Cathode

First, solution A was prepared by dissolving 5 mmol of FeCl_2_·4H_2_O in 100 mL of DI water, while solution B was prepared by dissolving 5 mmol of K_4_Fe(CN)_6_·3H_2_O, 10 mmol of potassium citrate, and 12 g of potassium chloride in 100 mL of DI water. Then, Solution A was slowly added to solution B with a dripping rate of 50 mL hr^−1^ under 400 rpm stirring for 4 h. The precipitates were collected by centrifugation and washed with the mixture of ethanol and DI water for 3 times, and finally dried in a vacuum oven for 2 h at 80 °C.

### Electrochemical Measurement

The electrochemical performances of the electrodes were evaluated using CR2032 coin‐type cells. A homogeneous slurry of anode material was prepared by mixing 70 wt.% of active material, 20 wt.% of NaCMC, and 10 wt.% of Super‐P in Deionized water. The mixture was coated on copper foil and then dried at 80 °C under Argon atmosphere. The average mass loading of active material for the half‐cell was ≈0.8–1.0 mg cm^−2^. K metal and glass fiber were used as counter electrode and separator, respectively. The electrolyte used in the cell was 1 m KFSI in DMC (≈160 µL per cell). The CV and EIS tests were collected by a multi‐channel electrochemical analyzer (Bio‐Logic‐science Instruments, VMP3). The electrochemical performances were tested on Neware battery analyzer (Neware, China) in the potential range of 0.01–3 V (vs K^+^/K). The galvanostatic GITT was evaluated by Maccor Series 4000 battery test system. For Mott–Schottky measurements, the CPS‐h at different cycles were employed as working electrodes, while Ag/AgCl and Pt were used as the reference and counter electrode, respectively. The Mott–Schottky plots were carried out in 0.5 m Na_2_SO_4_ aqueous solution at a constant frequency of 10 kHz.

### Potassium Ion Full Cells of CPS‐h/G||PB

For full cell assembly, PB was used as cathode material, and prepotassiated for 10 cycles at 250 mA g^−1^, while the anode material was also prepotassiated for 10 cycles at 500 mA g^−1^. The mass ratio of cathode to anode were 5.2:1. The whole assembly process was performed in an Argon‐filled glove box. The Galvanostatic discharge–charge tests were evaluated by NEWARE CT‐4000 battery measurement system from 1.0–3.8 V versus K^+^/K.

### Potassium Ion Hybrid Capacitors of CPS‐h/G||AC

For potassium ion hybrid capacitor assembly, the commercial AC was used as the cathode material, the CPS‐h/G was used as the anode and prepotassiated for 10 cycles. The mass ratio of cathode and anode was 1:1 and the working window was 0.5–4.0 V versus K^+^/K.

## Conflict of Interest

The authors declare no conflict of interest.

## Author Contributions

W.‐W.S. and H.‐Y.T. came up with the original idea and designed the experiments. W.‐W.S. and C.‐W.C. performed and analyzed the experiments. Y.‐Y.S. and Y.‐C.Y. helped the in situ XRD experiment. M.‐Y.L. and K.‐Y.H. helped the HAADF‐STEM experiments. Y.‐C.Y., W.‐W.S., and H.‐Y.T. wrote the paper. The study was conceived and supervised by H.‐Y.T.

## Supporting information

Supporting Information

## Data Availability

The data that support the findings of this study are available from the corresponding author upon reasonable request.
