# Supplementary material for: Thermodynamic Origin‐Based In Situ Electrochemical Construction of Reversible p‐n Heterojunctions for Optimal Stability in Potassium Ion Storage
Source: Adv Sci (Weinh). 2024 Mar 13;11(19):2308582. doi: 10.1002/advs.202308582 (PMC11109633; doi:10.1002/advs.202308582)
Supplement: Supplementary file 1 — Supporting Information [file ADVS-11-2308582-s001.pdf]

ADVANCED SCIENCE
Open Access

Supporting Information

for *Adv. Sci.*, DOI 10.1002/advs.202308582

Thermodynamic Origin-Based In Situ Electrochemical Construction of Reversible p-n Heterojunctions for Optimal Stability in Potassium Ion Storage

*Wei-Wen Shen, Yi-Yen Hsieh, Yi-Chun Yang, Kai-Yuan Hsiao, Ming-Yen Lu, Chi Wei Chou and Hsing-Yu Tuan**

Supporting Information

Thermodynamic Origin-Based *In Situ* Electrochemical Construction of Reversible p-n Heterojunctions for Optimal Stability in Potassium Ion Storage

Wei-Wen Shen, Yi-Yen Hsieh, Yi-Chun Yang, Kai-Yuan Hsiao, Ming-Yen Lu, Chi Wei Chou and Hsing-Yu Tuan[*]

W.-W. Shen, Y.-Y. Hsieh, Y.-C. Yang, C.-W. Chou, H.-Y. Tuan

Department of Chemical Engineering, National Tsing Hua University, Hsinchu 30013, Taiwan

Email: hytuan@che.nthu.edu.tw

K.-Y. Hsiao, M.-Y. Lu

Department of Materials Science and Engineering, National Tsing Hua University, Hsinchu 30013, Taiwan

Experimental Section

Materials

Commercial copper particles (less than 425 µm, 99.5 % purity), Potassium metal (98%), Selenium powder (~325 mesh, 99.5%, metal basis), dimethyl carbonate (DMC, anhydrous 99 %), NaCMC (average MW ≈700 000), potassium hexacyanoferrate (II) trihydrate ($K_4Fe(CN)_6 \cdot 3H_2O$, 98.5%), potassium chloride (KCl, 99%), ethanol (99.5%),chloride (CuCl, 99%), Hydrazine ($N_2H_4 \cdot H_2O$), and ethylene glycol (EG, 99%) were purchased from Sigma Aldrich. Red phosphorus powder (P, 98.9%, amorphous, metal basis), selenium oxide (SeO_2, 99.4%) and Iron (II) chloride tetrahydrate ($FeCl_2 \cdot 4H_2O$, 98%) were purchased from Alfa Aesar. Potassium bis (fluorosulfonyl)imide (KFSI, 97 %) was purchased from Combi-Blocks. Super-P, graphite, and coin-type cell CR2032 were purchased from shining energy. Glass fiber was purchased from Advantec. Copper foil were purchased from Chang-Chun group. All the chemicals were used without purification.

Material Characterization

The crystal purity was determined by an X-ray diffractometer (XRD, Bruker D8 ADVANCE) with a Cu-Kα source (λ = 1.54056 Å). The morphologies of the obtained samples were examined by employing scanning electron microscopy (SEM, HITACHI-SU8010) and an energy dispersive spectrometer (EDS) was used for elemental mapping. Transmission electron microscopy (TEM, JEOL, JEM-ARM200FTH, serviced provided by NTHU) with an accelerating voltage of 200 kV for investigating structural analysis including morphology, crystal d-spacing, and selected-area electron diffraction (SAED). The chemical state was examined by high-resolution X-ray photoelectron spectroscopy (XPS, ULVAC-PH, PHI QuanteraII). All the spectra obtained from the XPS analysis were first calibrated by referencing the standard binding energy of C 1s

(284.8 eV). Raman spectrum was measured via a LABRAM HR 800 UV with a 532 nm excitation source. TGA analysis was obtained using a thermogravimetric analyzer (TA, Q50) in an air flow at a heating rate of 10 °C min^{-1} from ambient temperature to 800 °C. The UPS (ULVAC PHI 5000 Versa Probe, serviced provided by NTU). The UV-Vis-DRS (Jasco V-770, serviced provided by Precision Research and Analysis Center, NTUT). The Scanning Probe Microscopy system (Bruker Dimension ICON) was provided by The Instrumentation Center at NTHU.

DFT calculations

First-principle calculation was performed in the framework of density functional theory as implemented in the VASP program.[1] The generalized gradient approximation proposed by Perdew, Burke, and Ernzerhof is selected for the exchange-correlation potential.[2] The energy criterion is set to 10^{-4} eV in iterative solution of the Kohn-Sham equation. For all models the vacuum space along the z-direction was set to be 15 Å, which was enough to avoid interaction between the two neighboring images. The M-P Brillouin zone sampling grid of spacing $2\pi \times 0.03$ $Å^{-1}$ and a cutoff energy of 500 eV was employed. All the structures are relaxed until the residual forces on the atoms have declined to less than 0.02 eV/Å. The barriers for K^+ diffusion are calculated with the climbing-image nudged elastic band (CI-NEB) method.[3] The MD simulations were performed using VASP program. A minimal Γ-centered 1×1×1 k-point grid was used to keep the computational cost at a reasonable level. The system is equilibrated at the equilibrium temperature (300K and 500K) for 10 ps at constant volume with a Nosé thermostat. The time step was chosen to be 2 fs, and the integration of Newton's equation is based on the Verlet algorithm implemented in VASP.

Bader charge analysis

The interfacial electron transfer of CPS-h was calculated using a Bader analysis program based on the CuSe(110) and PSe($\bar{1}10$) surfaces from the HAADF-STEM

images.

Table S1. Comparison of D_{K}^{+} values of CPS-h/G full cell in this work with previously reported.

Material	D_{K+} value	Ref.
$CoSe_2/FeSe_2$@C	about 5×10^{-10} to 2×10^{-9}	[4]
$Ni_3Se_4/CoSe_2$@C	about 1×10^{-14} to 1×10^{-12}	[5]
Cu_9S_5-MoS_2	about 1×10^{-12} to 3×10^{-11}	[6]
$CoSe_2$-Cu_2Se@NC	about 5×10^{-12} to 1×10^{-14}	[7]
Bi_2S_3/Bi_2Se_3 vdWHs	about 1×10^{-12} to 1×10^{-9}	[8]
Ni-doped $FeSe_2/Fe_3Se_4$ heterojunction	1×10^{-9} to 1×10^{-7}	[9]
$CoSe_2$-$FeSe_2$/graphite/N-doped carbon	3×10^{-11} to 2×10^{-10}	[10]
$MoSe_2$@MoO_2	1×10^{-11} to 1×10^{-9}	[11]
**This work**	4.35×10^{-11} to 7.23×10^{-10}	

Table S2. Net charge of Cu, P, Se atoms in CPS-h.

Element	Net charge
Cu	4.3524
P	−1.87746
Se	−2.47494

Table S3. Summary of the electrochemical performance between potassium-ion battery and hybrid capacitors for the CPS-h anode.

System	Cathode	Working voltage (V)	Cycle number	Energy density (Wh kg^{-1})	Power density (W kg^{-1})
**Potassium-ion battery**	Prussian blue	1.0–3.8 V	2000 cycles at 1 A g^{-1}	101.35-135.79	323-13698
**Hybrid capacitors**	activated carbon	0.5–4V	4000 cycles at 1.5 A g^{-1}	60.32-88.42	207-3877

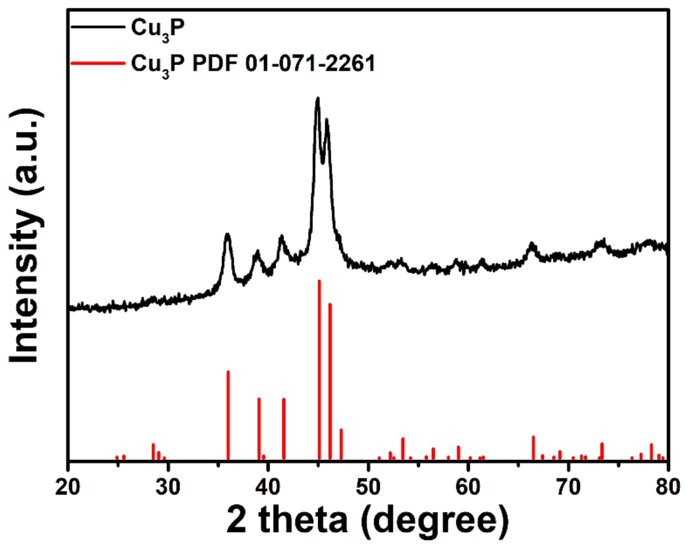

Figure S1. XRD pattern of Cu_3P.

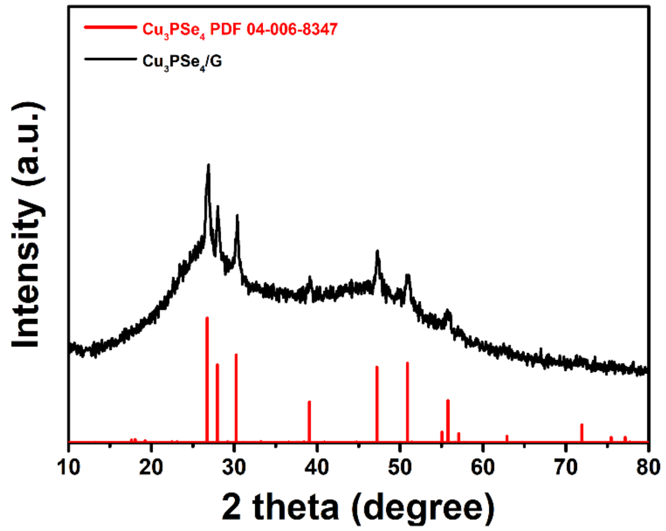

Figure S2. XRD pattern of Cu_3PSe_4/G.

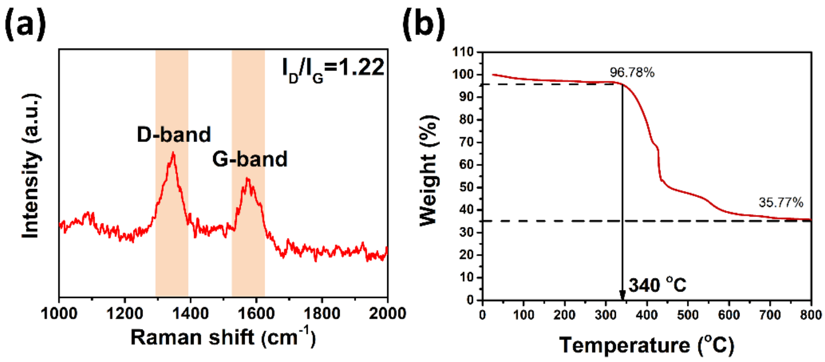

Figure S3. (a) Raman spectra of Cu_3PSe_4/G. (b) TGA curve of Cu_3PSe_4/G.

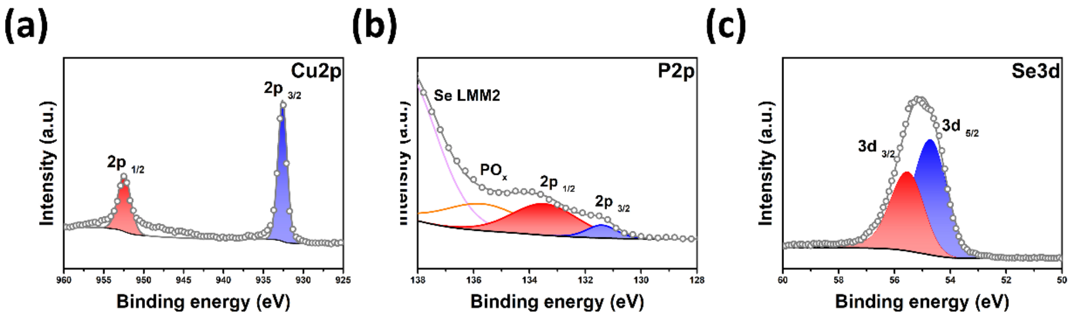

Figure S4. XPS spectra of Cu_3PSe_4 powder.

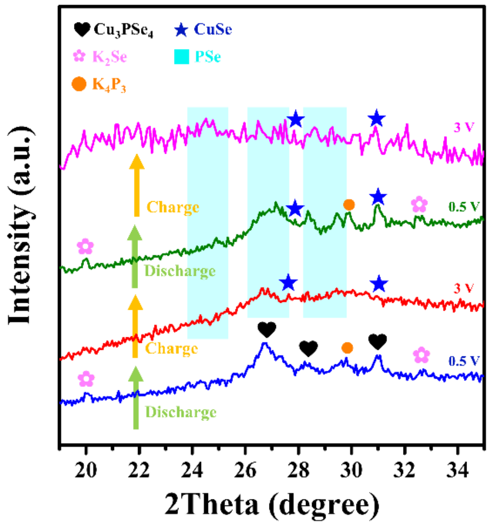

Figure S5. Ex situ XRD patterns of Cu_3PSe_4 for first two cycles.

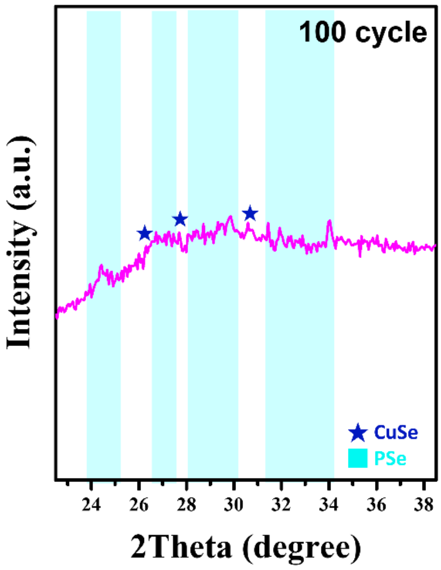

Figure S6. XRD pattern of Cu_3PSe_4 after 100 cycles.

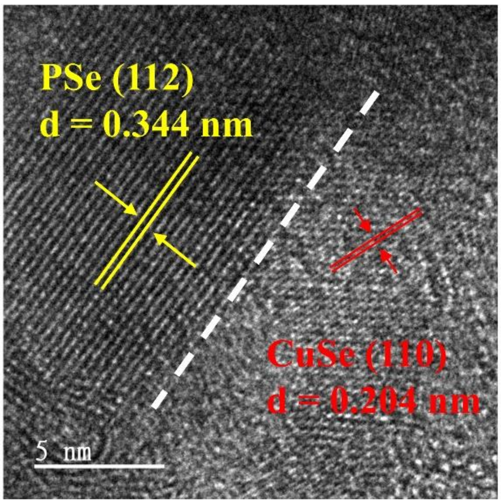

Figure S7. HRTEM image of Cu_3PSe_4 after 100 cycles.

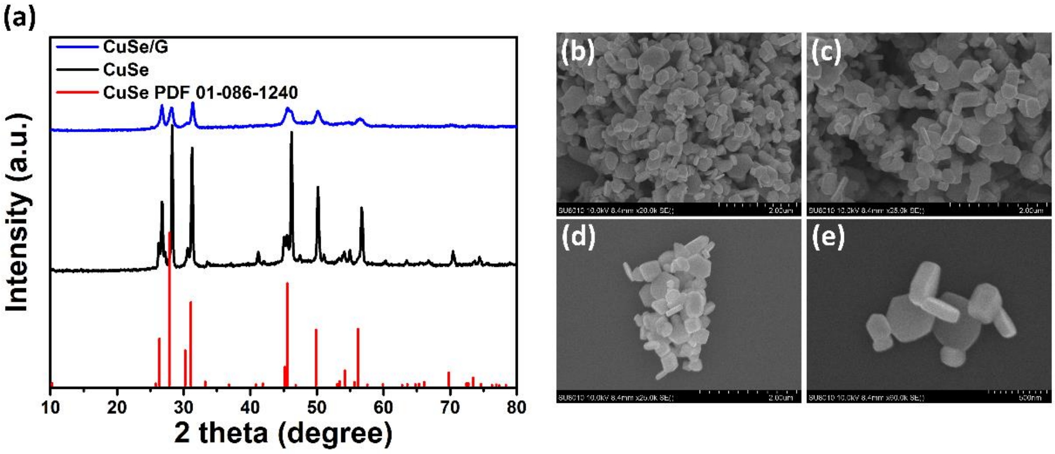

Figure S8. (a) XRD pattern and (b) SEM images of CuSe.

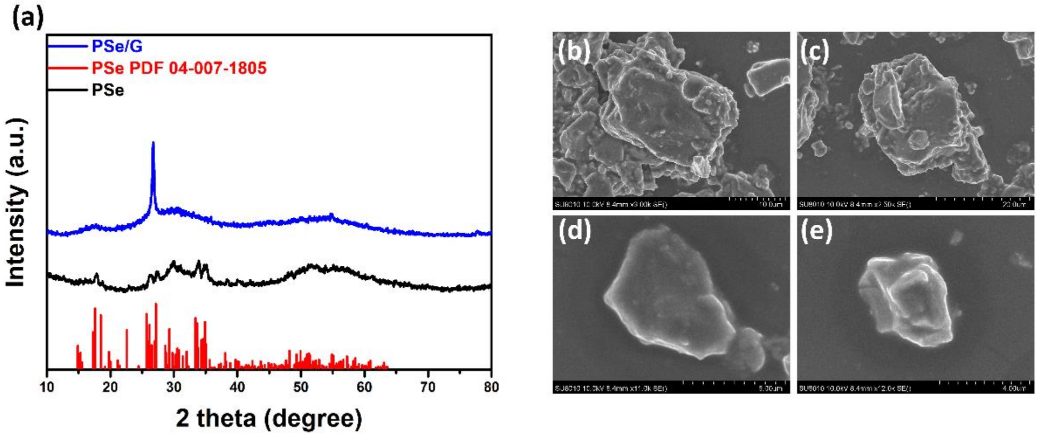

Figure S9. (a) XRD pattern and (b) SEM images of PSe.

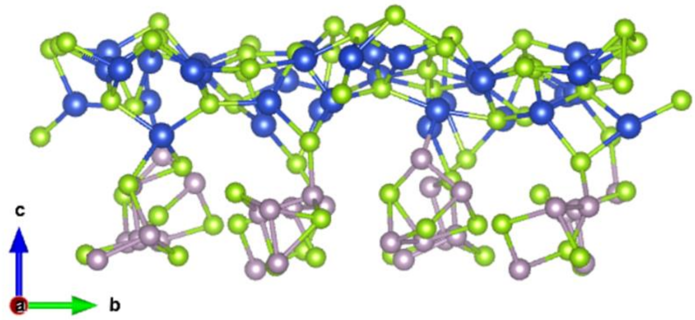

Figure S10. The unit cell structure of CuSe/PSe heterostructure (CPS-h).

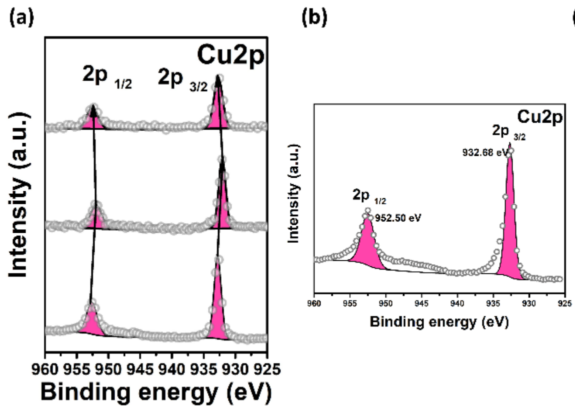

(c)

	Cu 2p 1/2 (eV)	Cu 2p 3/2 (eV)
OCV	952.60	932.80
D0.01V	951.92	932.08
C3V	952.47	932.65
CuSe	952.50	932.68

Figure S11. (a) *Ex situ* XPS spectra of Cu 2p at first cycle. (b) Cu 2p spectra of CuSe powder. (c) The binding energies of Cu 2p $_{1/2}$ and Cu 2p $_{3/2}$.

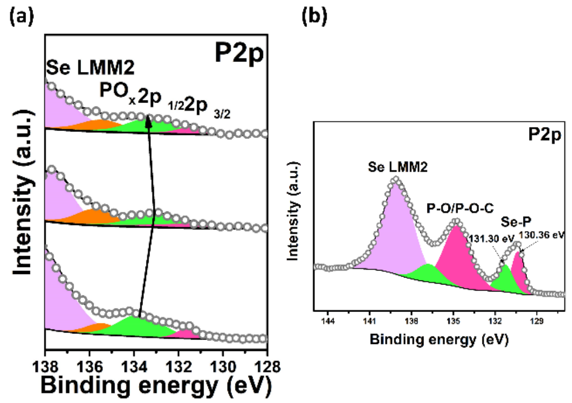

(c)

	P $2p_{1/2}$ (eV)	P $2p_{3/2}$ (eV)
OCV	133.72	131.46
D0.01V	133.11	131.26
C3V	133.25	131.42
CuSe	131.30	130.36

Figure S12. (a) *Ex situ* XPS spectra of P 2p at first cycle. (b) P 2p spectra of PSe powder. (c) The binding energies of P 2p $_{1/2}$ and P 2p $_{3/2}$.

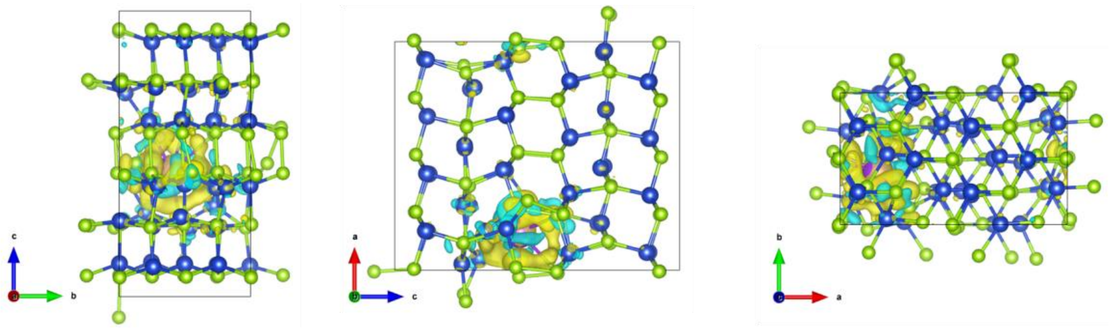

Figure S13. Charge density difference of CuSe with K-ion adsorbed.

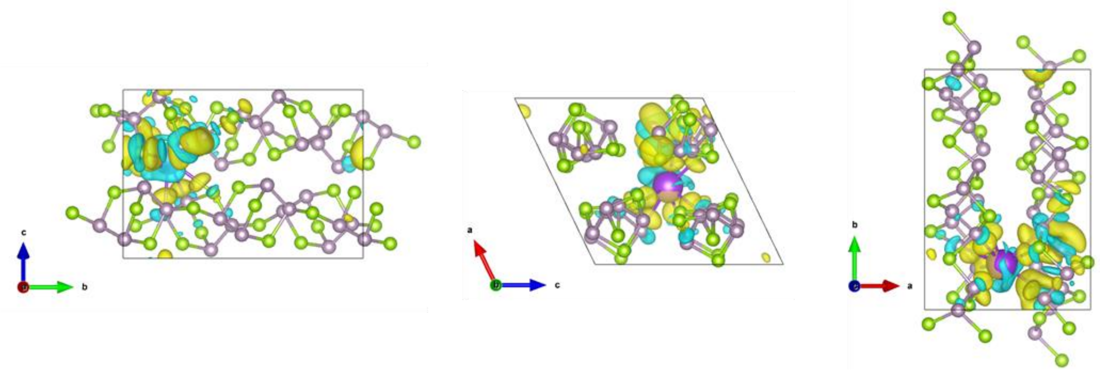

Figure S14. Charge density difference of PSe with K-ion adsorbed.

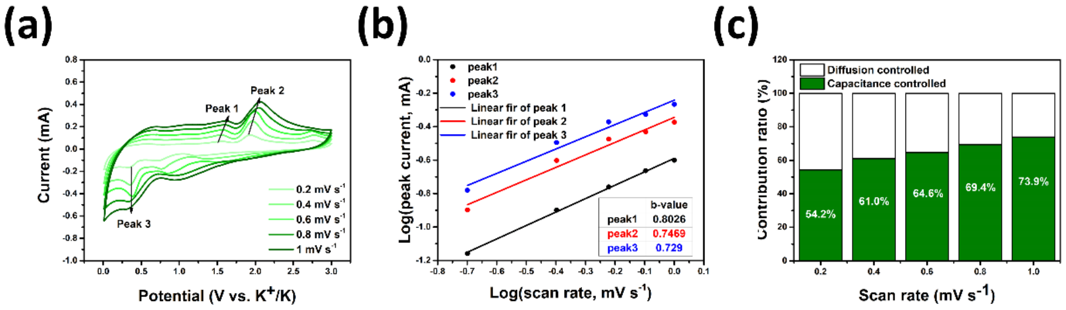

Figure S15. (a) CV curves at various scan rates of CuSe/G. (b) Linear fitting profiles of log (i, peak current) vs log (v, scan rate) of CuSe/G. (c) Capacitance contribution of CuSe/G.

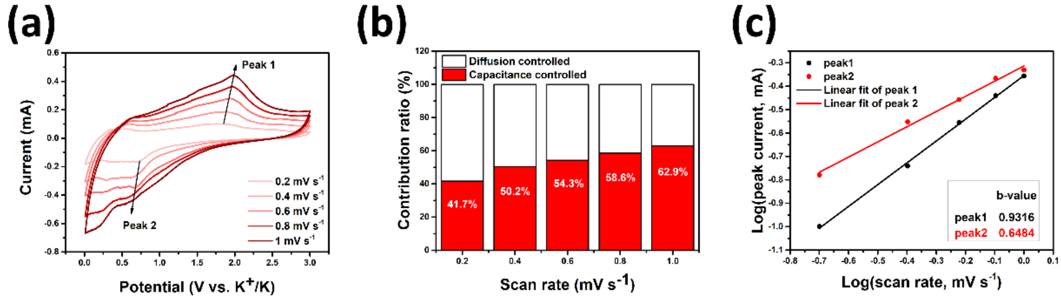

Figure S16. (a) CV curves at various scan rates of PSe/G. (b) Linear fitting profiles of log (i, peak current) vs log (v, scan rate) of PSe/G. (c) Capacitance contribution of PSe/G.

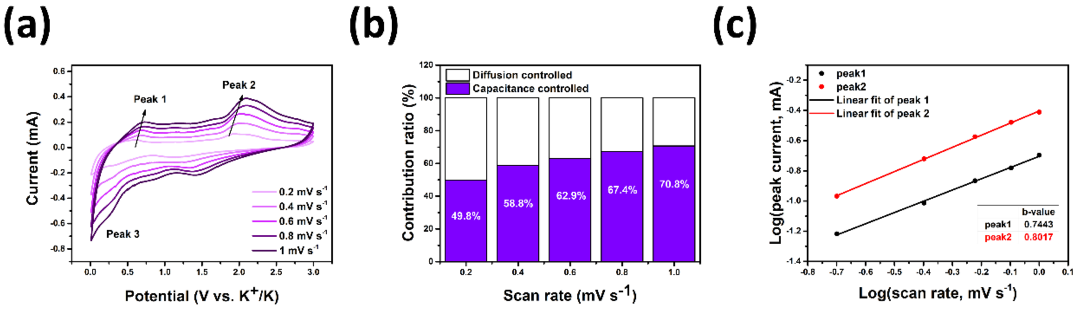

Figure S17. (a) CV curves at various scan rates of CPS/G. (b) Linear fitting profiles of log (i, peak current) vs log (v, scan rate) of CPS/G. (c) Capacitance contribution of CPS/G.

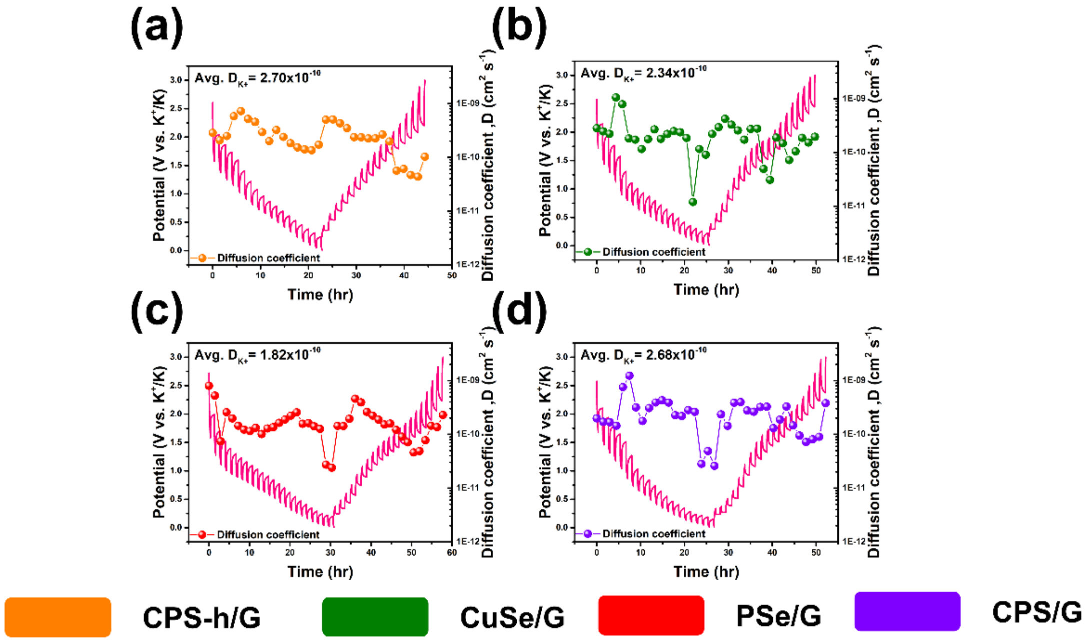

Figure S18. GITT curves and diffusion coefficient of (a) CPS-h/G, (b) CuSe/G, (c) PSe/G, and (d) CPS/G.

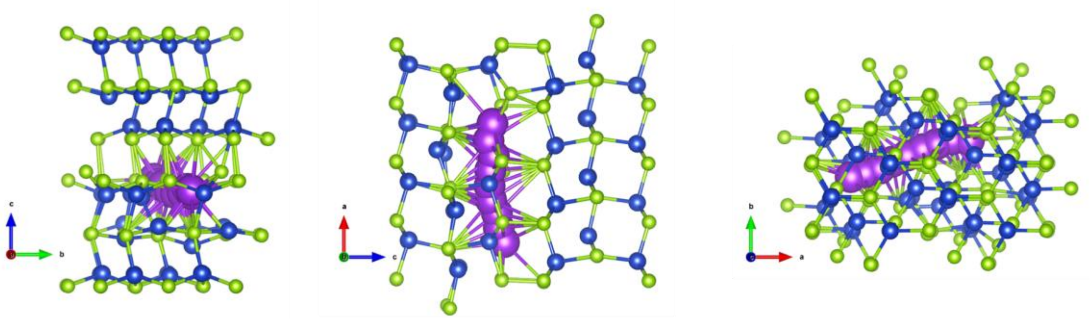

Figure S19. K-ion diffusion path in CuSe.

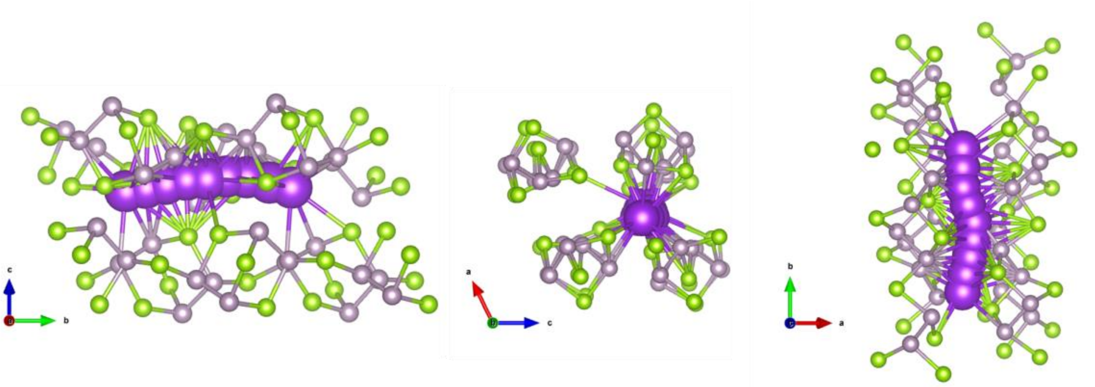

Figure S20. K-ion diffusion path in PSe.

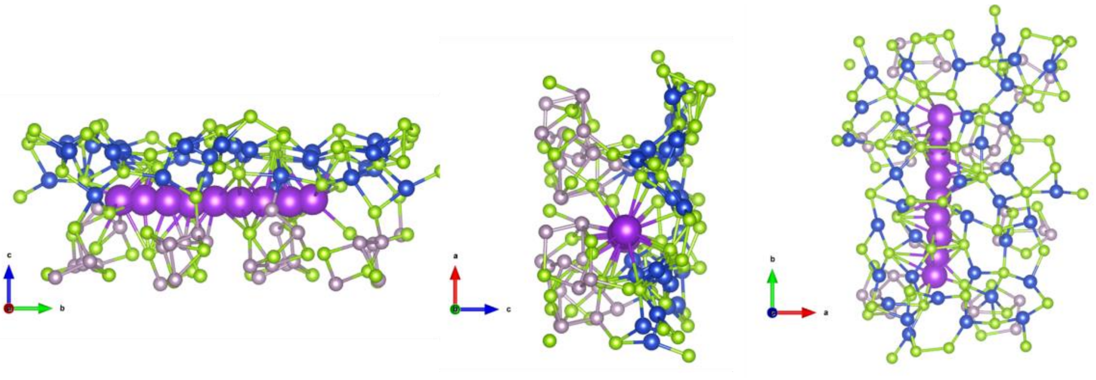

Figure S21. K-ion diffusion path in CPS-h.

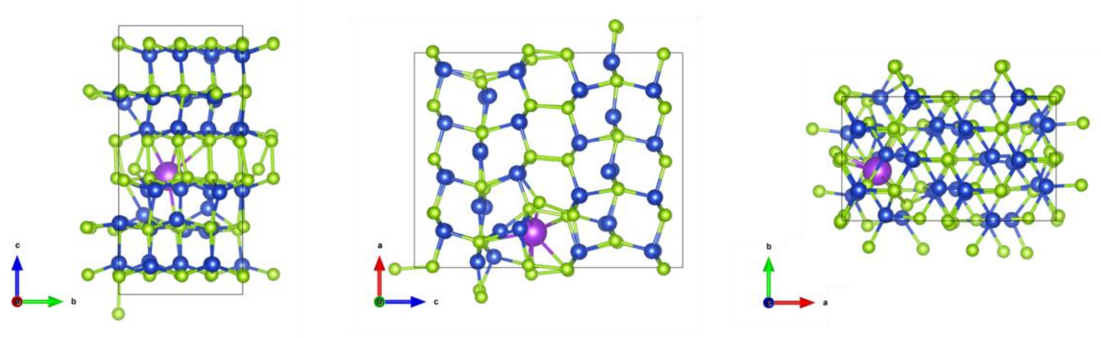

Figure S22. CuSe structure with K-ion adsorbed.

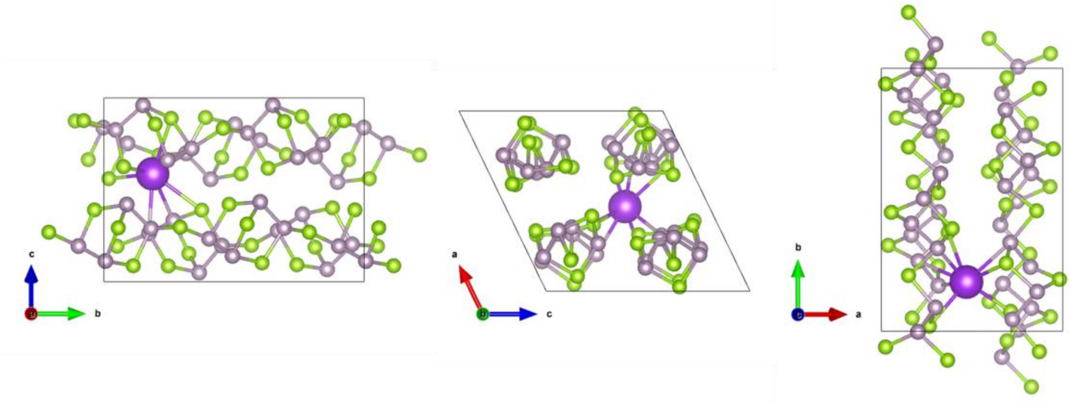

Figure S23. PSe structure with K-ion adsorbed.

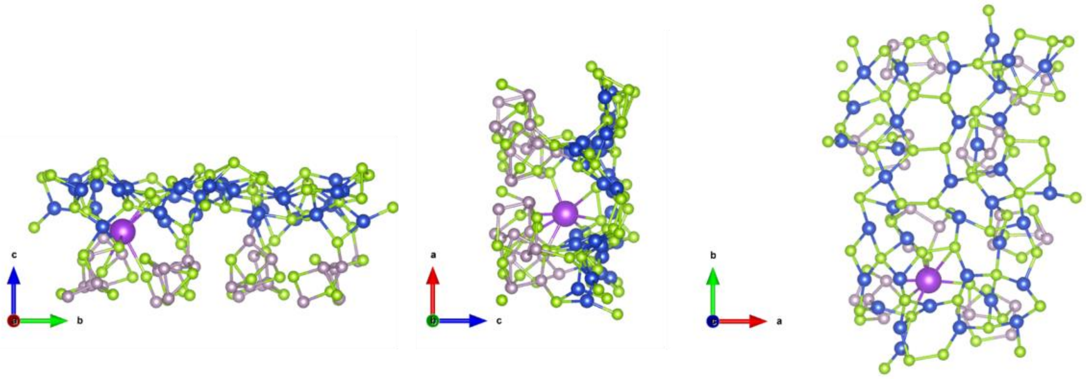

Figure S24. CPS-h structure with K-ion adsorbed.

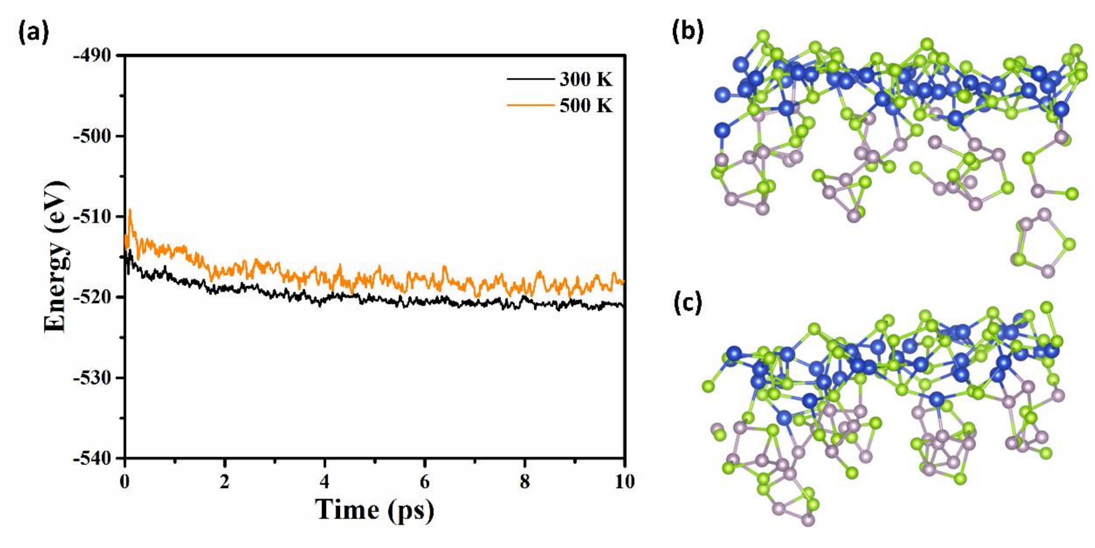

Figure S25. (a) The potential energy of the CPS-h from the AIMD simulation at 300 K and 500 K. (b, c) Side view of the final configurations of the CPS-h heterostructure at 300 K and 500 K.

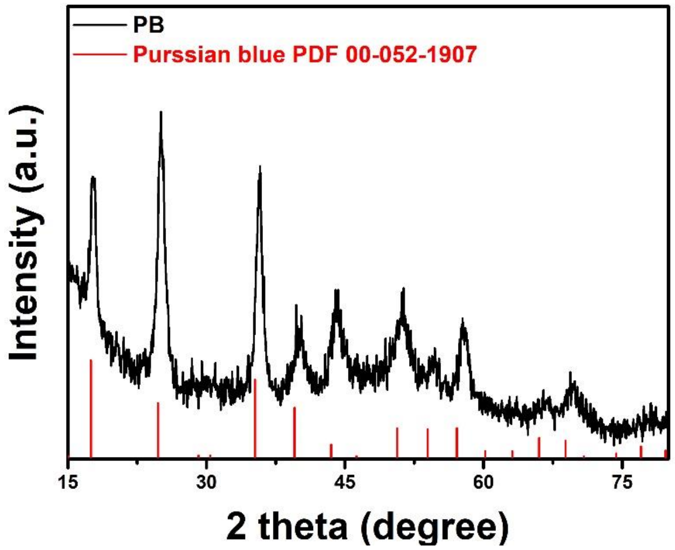

Figure S26. XRD pattern of PB cathode.

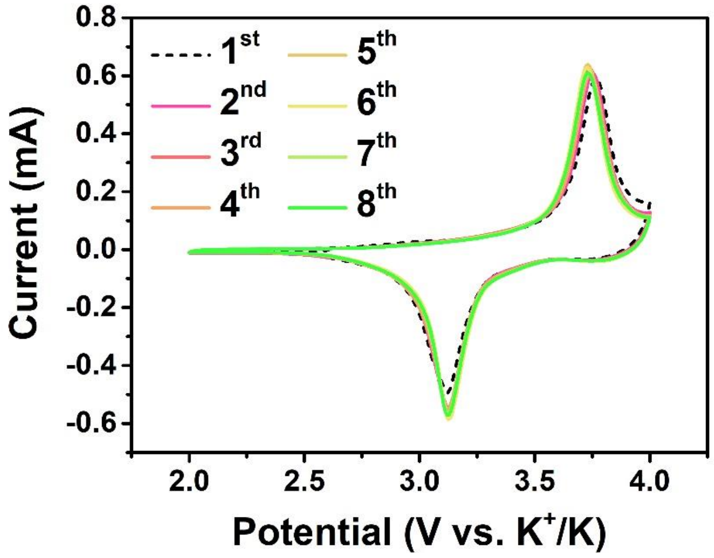

Figure S27. CV curves of PB half cell.

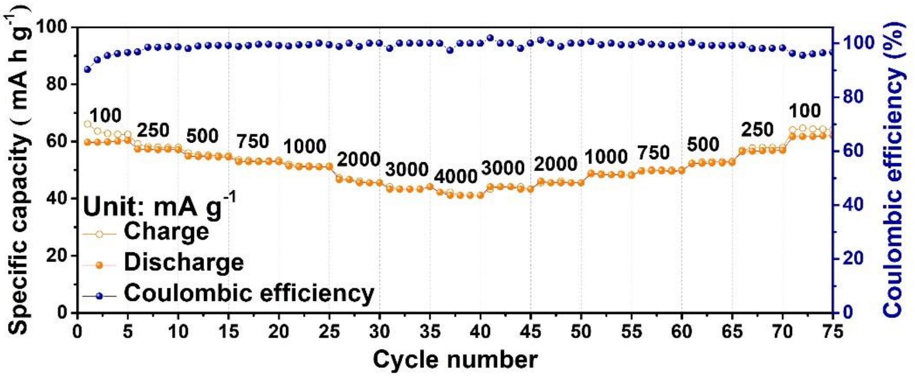

Figure S28. Rate performance of PB half cell at various current densities.

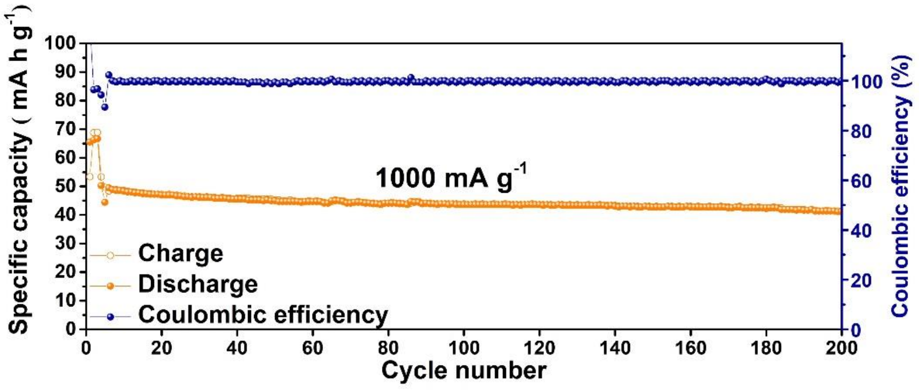

Figure S29. Long-term cycling performance of PB half cell at 1000 mA g^{-1}.

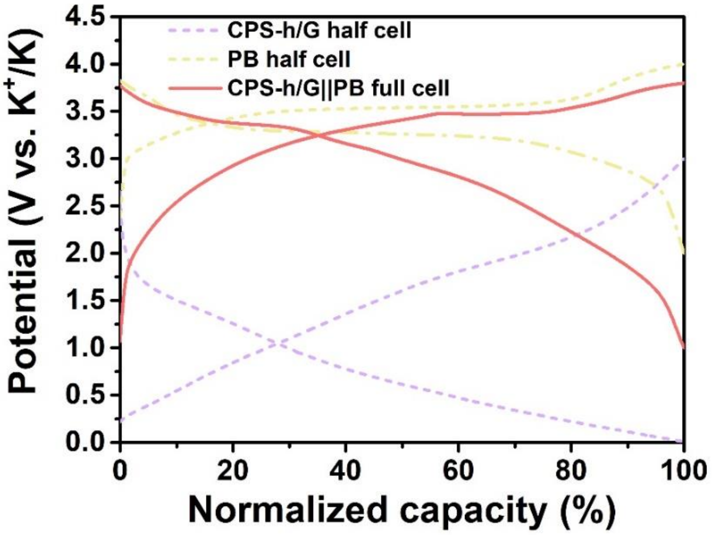

Figure S30. The normalized GCD curves of a half cell and a CPS-h/G||PB full battery.

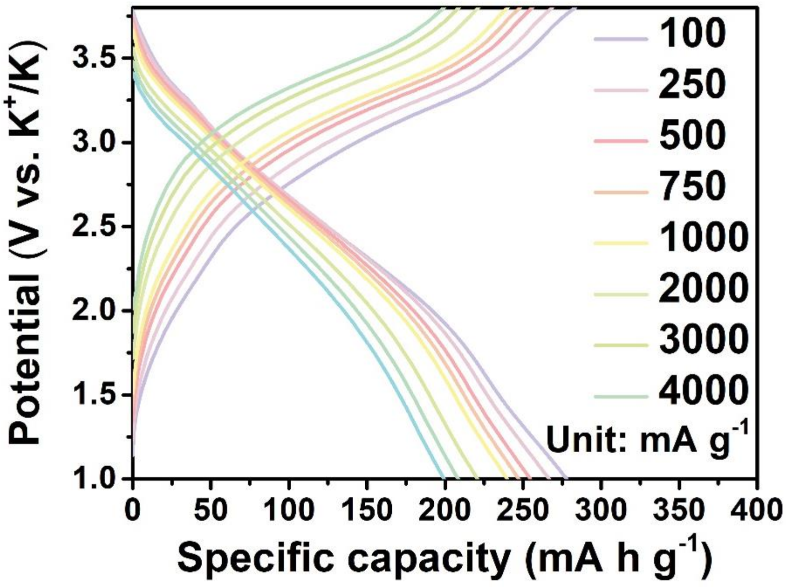

Figure S31. GCD curves at different current densities ranging from 0.1 to 4 A g^{-1}.

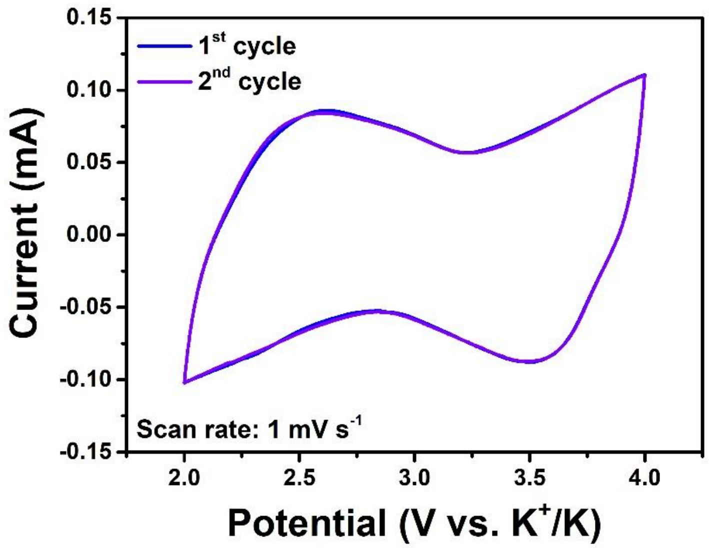

Figure S32. CV curves of AC half cell at 1 mV s $^{-1}$.

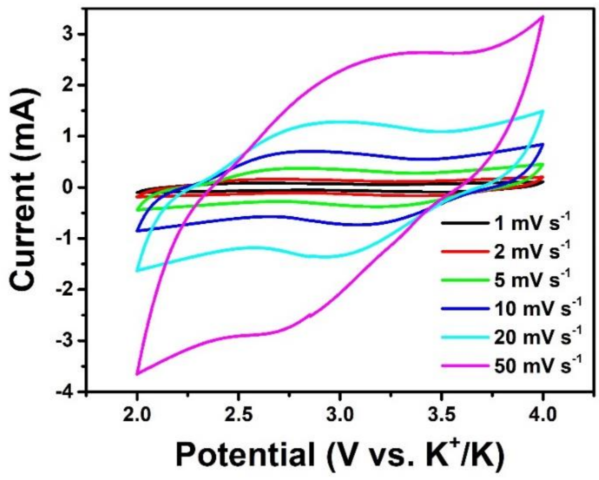

Figure S33. CV curves of AC half cell at various scan rates.

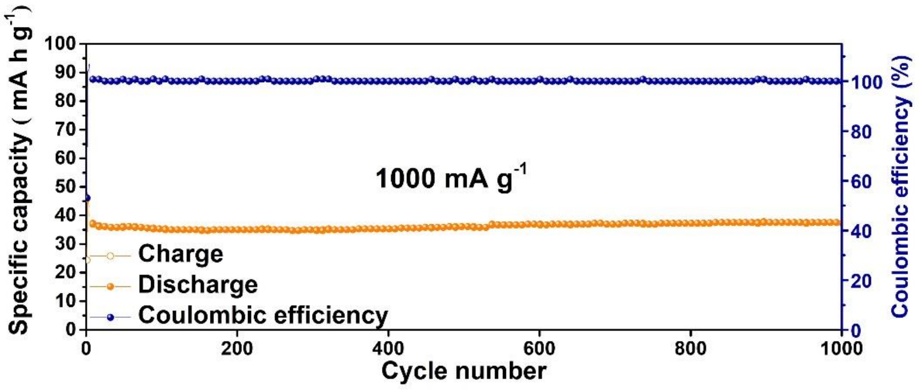

Figure S34. Long-term cycling performance of AC half cell at 1000 mA g^{-1}.

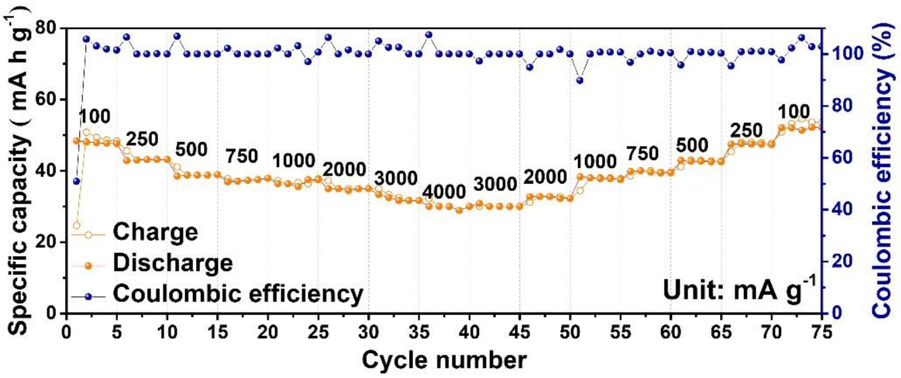

Figure S35. Rate performance of AC half cell at various current densities.

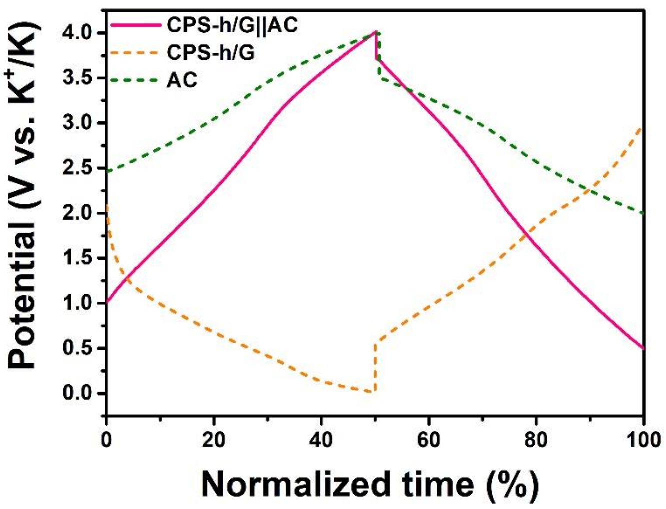

Figure S36. Normalized GCD curves of a half cell and a full cell.

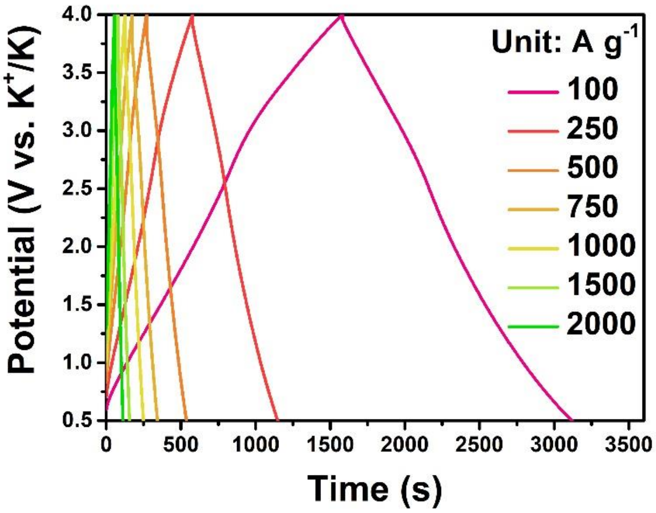

Figure S37. GCD curves at the current densities ranging from 100 to 2000 mA g^{-1}.

References

[1] G. Kresse, D. Joubert, *Phys. Rev. B* **1999**, *59*, 1758.

[2] J. P. Perdew, K. Burke, M. Ernzerhof, *Phys. Rev. Lett.* **1996**, *77*, 3865.

[3] G. Henkelman, B. P. Uberuaga, H. Jónsson, *J. Chem. Phys.* **2000**, *113*, 9901.

[4] H. Shan, J. Qin, Y. Ding, H. M. K. Sari, X. Song, W. Liu, Y. Hao, J. Wang, C. Xie, J. Zhang, *Adv. Mater.* **2021**, *33*, 2102471.

[5] Y. Zhang, S. Wei, Z. Zhao, X. Pei, W. Zhao, J. Wang, X. Du, D. Li, *Small* **2022**, *18*, 2107258.

[6] B. Zhang, B. Xu, H. Qin, L. Cao, X. Ou, *J. Mater. Sci. Technol.* **2023**, *143*, 107.

[7] X. Liu, Z. Niu, Y. Xu, Z. Zhao, C. Li, Y. Yi, H. Guan, S. Zhang, X. Pei, D. Li, *Chem. Eng. J.* **2022**, *430*, 133176.

[8] Y.-Y. Hsieh, H.-Y. Tuan, *Energy Storage Mater.* **2022**, *51*, 789.

[9] Z. Kong, L. Wang, S. Iqbal, B. Zhang, B. Wang, J. Dou, F. Wang, Y. Qian, M. Zhang, L. Xu, *Small* **2022**, *18*, 2107252.

[10] S.-S. Mai, K.-Y. Hsiao, Y.-C. Yang, Y.-R. Lu, M.-Y. Lu, Y.-Y. Hsieh, C.-B. Chang, H.-Y. Tuan, *Chem. Eng. J.* **2023**, *474*, 145992.

[11] Q. Jiang, S. Hu, L. Wang, Z. Huang, H.-J. Yang, X. Han, Y. Li, C. Lv, Y.-S. He, T. Zhou, *Appl. Surf. Sci.* **2020**, *505*, 144573.
